# Quality assurance of online adaptive radiotherapy workflows using film dosimetry in a 3D printed thorax anthropomorphic phantom

**DOI:** 10.1016/j.phro.2026.100909

**Published:** 2026-01-22

**Authors:** Daan Hoffmans, Koen Nelissen, Eva Versteijne, Wilko Verbakel

**Affiliations:** a Amsterdam UMC Location Vrije Universiteit Amsterdam, Department of Radiation Oncology, De Boelelaan 1117, Amsterdam, the Netherlands; b Cancer Center Amsterdam, Cancer Treatment and Quality of Life, Amsterdam, the Netherlands; c Varian Medical Systems, Radiotherapy Solutions, Palo Alto, USA

**Keywords:** 3D printing, Anthropomorphic phantom, Online adaptive radiotherapy, End-to-end, QA

## Abstract

**Background and purpose:**

Quality Assurance for online adaptive radiotherapy (oART) can be challenging. Several tests can demonstrate the dosimetric and position accuracy, but commercial phantoms are often not anatomically representative. The aim of this study was to investigate the accuracy of cone-beam computed tomography guided oART palliative and breast cancer trials by using a 3D printed thorax anthropomorphic phantom.

**Materials and methods:**

An anthropomorphic phantom was 3D printed for this study which accommodates film through the spine, breast, heart, and lungs. Dose was measured for spine and breast treatment plans, whilst variations were simulated which can occur during treatment. Measurements were compared to calculated dose on the planning (pCT) and synthetic computed tomography (sCT) using gamma pass rate criteria of minimal 95  % (for gamma of 4  %/2 mm). Differences between the mean gamma were tested for significance.

**Results:**

Measurements done with positional and target volume changes showed no significant difference between the gamma analyses for the pCT and sCT (p = 0.15), indicating a robust and safe workflow. For extreme variations, difference was found between gamma analyses for the pCT and sCT (p = 0.051). Pass rates were all >95  %, except for three measurements in which the sCT showed density errors up to 1000 Hounsfield Units.

**Conclusions:**

This QA approach for oART, which used film measurements in a custom 3D-printed anthropomorphic phantom was able to validate the accuracy of the oART workflow when anatomical deviations arise and could be suitable as end-to-end test in the future.

## Highlights

• End-to-end test with an anthropomorphic phantom in adaptive radiotherapy is feasible.
• For 30 film measurements, 24 had gamma pass-rates of >95 % with 4 % / 2 mm.
• The end-to-end test was sensitive for deviating densities (∼1000 Hounsfield Units).
• The online adaptive workflow was deemed safe and within clinical criteria.

## Graphical abstract

**Figure:**
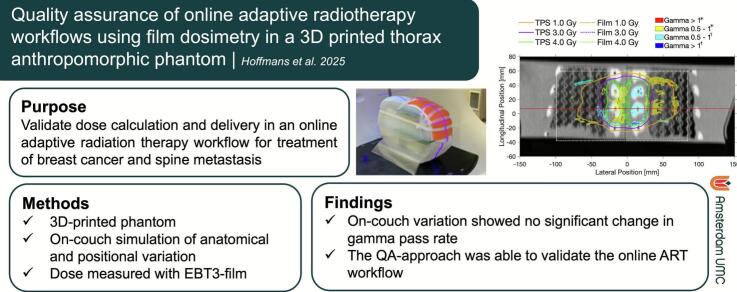

## 1 Introduction

Recent developments in online adaptive radiotherapy (oART) have led to an increase in patients treated with a treatment plan optimized based on the daily anatomy while the patient is positioned on the treatment couch [1], [2], [3], [4]. For both Magnetic Resonance (MR)-guided and cone-beam computed tomography (CBCT)-guided adaptive RT, quality assurance (QA) can be complex due to the nature of these procedures [[5], [6], [7], [8], [9], [41]]. One challenge for oART is that measurement-based patient specific QA (PSQA) can only be performed after or during (in-vivo) treatment, since the treatment plan is optimized while the patient is on the treatment couch. Additionally, the oART workflow is a chain consisting of many factors such as positioning, deformation, density propagation, optimization, calculation etc. This chain is not covered in most QA measurements. End-to-end tests more thoroughly evaluate the accuracy of a patient treatment for the complete process by quantifying the dosimetric and geometric accuracy through a measurement in a phantom. Therefore, end-to-end tests should be part of the commissioning process of a new oART workflow [[6], [41]].

For end-to-end testing, various types of commercial phantoms are available [10], [11], [12], [13] which often are limited in patient resemblance, anatomical region, and flexibility in detector placement. Some commercial phantoms can accommodate anthropomorphic inserts [5], [12], [13], [14] tailored to a treatment site [15], [16], [17], or specifically for MR-guided radiotherapy [18], [19], [20], [21], [22]. Phantoms for breast are limited in literature [12], [23]. In the last decade, 3D printing techniques allowed development of phantoms with more realistic anthropomorphic geometry and densities [24], [25].

For this study, we used a 3D printed anthropomorphic phantom, adapted from an earlier version [24], for film-based QA of CBCT-guided oART, and show its potential for end-to-end testing. We used breast cancer and fast palliative oART workflows to test dosimetric accuracy on an anthropomorphic phantom. Measurements were performed while simulating different positioning and anatomical changes by varying the orientation of the phantom, the extension of the target during the on-couch procedure or by using a treatment plan that was made on a CT from a different patient.

## 2 Materials and methods

### 2.1 Phantom construction

**Fig. 1:**
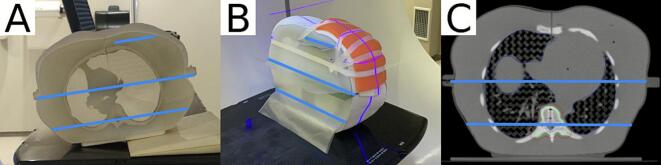
The 3D printed thorax anthropomorphic phantom, blue lines show film measurement planes: (A) phantom on couch with a rotation during CT acquisition, (B) phantom on-couch with added bolus, (C) CT of the phantom. (For interpretation of the references to colour in this figure legend, the reader is referred to the web version of this article.)

Similar to previous work, an anthropomorphic upper thorax phantom was 3D-printed based on the CT scan (Discovery CT590 RT, General Electric, Boston, USA) of a female patient [24]. The phantom (Fig. 1) was manufactured in six parts, which allowed the insert of dosimetric film in three coronal planes through the spine, the middle of the phantom and the left breast (Supplementary section A) [24].

### 2.2 Treatment planning and oART workflow

In this research we investigated the accuracy of oART for spine and breast [26], [27]. Treatment planning and delivery was performed in the Ethos system (Varian a Siemens Healthineers company, Palo Alto, USA). Treatment plans were generated on planning CT-scans according to standard departmental protocol, with 2.5  mm slice thickness. To emulate changes in anatomy or positioning in patients, the phantom was scanned in three different alignments: (1) Regular positioning; (2) A 6 degree pitch along the lateral axis; (3) A 6 degree pitch around the lateral axis and a 4 degree rotation around the vertical axis. Such differences in positioning can occur in our clinical diagnostic CT based oART workflow [26]. Additionally, this allowed investigation of a worst-case scenario whilst also forcing the system to perform re-optimization.

The CT-scans were sent to Aria (V16.00.00, Varian) for contouring. On each scan, a clinical target volume was defined as 1 vertebra and the whole breast left. For each target, a 5  mm planning target volume margin was used. The lungs, spinal canal + 3 mm, and heart were contoured as organs at risks (OARs).

**Fig. 2:**
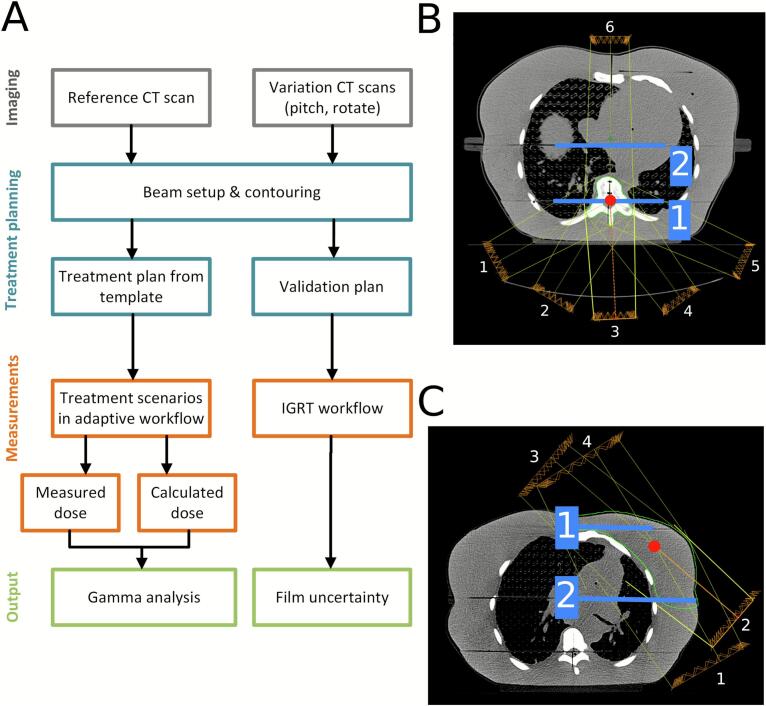
(A) the measurement workflow used, (B) the beam setup for spine, beams 1–6 depicted in white numbers, beam isocenter is shown with a red circle, and the dose planes 1–2 with a blue line. (C) the tangential beam setup of beams 1–4 for breast, the beam isocenter is shown with a red circle, and the two film panes with blue lines. (For interpretation of the references to colour in this figure legend, the reader is referred to the web version of this article.)

The beam setup was done in Eclipse (V16.1, Varian). For the spine, a posterior Intensity-Modulated Radiation Therapy (IMRT) beam setup (Fig. 2B) was used, delivering 4  Gy/fraction. For the breast, a scripted four-beam IMRT with tangential setup (Fig. 2C) was used, delivering 5.20  Gy/fraction. These plans were used for the determination of the film accuracy in measurements S0 and B0, and also exported to the Ethos treatment planning system (TPS) (V1.1MR1, Varian) for plan-optimization using clinical planning templates for breast and spine treatments (Supplementary section B).

The online adaptive workflow for the Ethos has been previously described in detail [28], [29], [30]. In short, the system generated a synthetic CT (sCT) based on a deformable registration between the planning CT (pCT) and CBCT, ensuring correct density information from the planning CT and daily anatomical accuracy for that specific treatment session. Each session, the Ethos optimized a new adapted treatment plan based on a template that is defined in the pre-treatment treatment planning, calculated the dose distribution on the sCT (which is visualized on the CBCT), and also calculated the dose of the scheduled (non-adapted) treatment plan on the sCT. During the online procedure, PSQA was performed by means of a secondary dose calculation by an independent dose calculation engine (Mobius, V4.1 Varian). The secondary dose calculation was compared to the clinical dose calculation using gamma criteria of 3  % global dose difference, 3  mm distance to agreement (3  %/3 mm) with a low dose threshold of 10  %.

### 2.3 Test scenarios

**Fig. 3:**
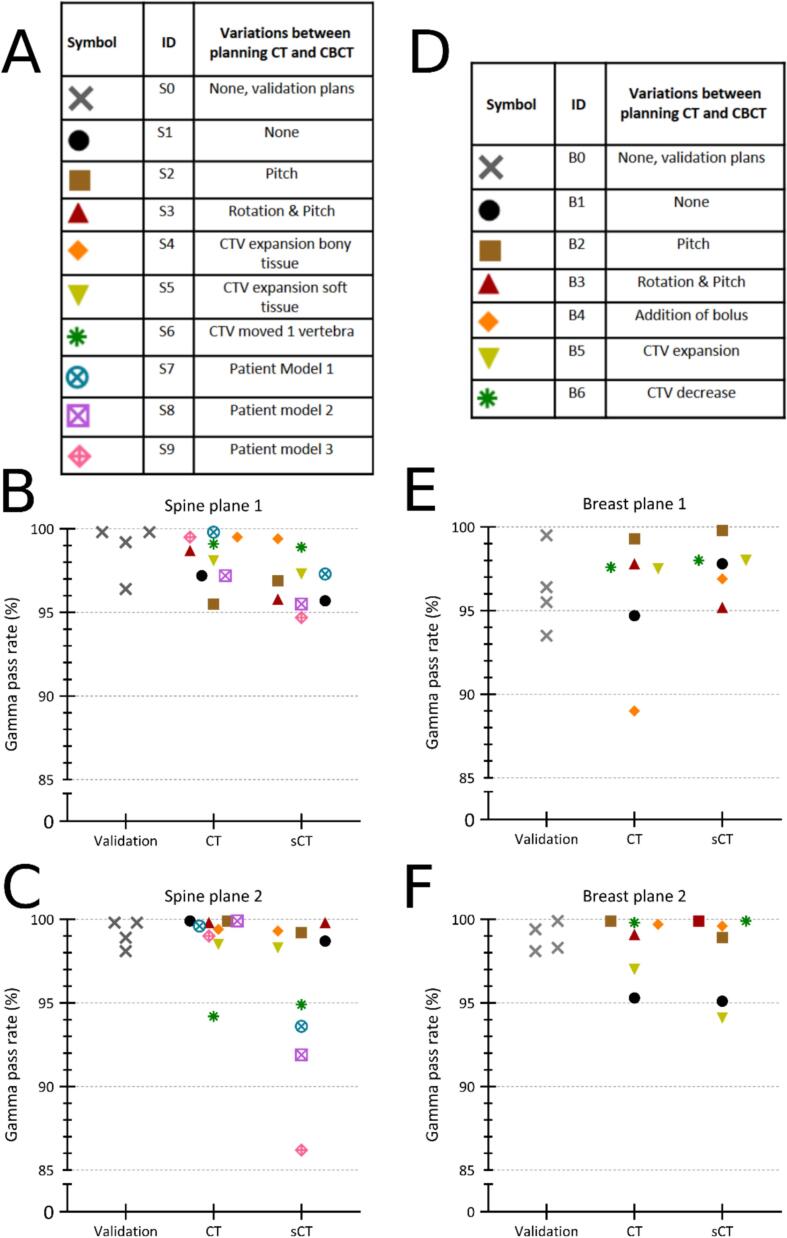
Phantom measurements spine and breast: (A,D) all measured scenarios and their corresponding symbols. Gamma pass rates for film measurements done in plane 1 (B,E) and plane 2 (C,F) for spine and breast respectively. *Abbreviation: sCT = synthetic CT.*

Various scenarios were measured to simulate changes, compared to pCT, in the patient's position or anatomy. The variations applied in the tests were phantom rotations, CTV shift or expansions or body expansion, described in more detail in Fig. 3A, 3D and Supplementary section C. All measurements for spine (SX) and breast (BX) were done using the adapted treatment plan, except for S1 and B1 where the scheduled plan was used for both sites to also test this part of the workflow. In measurement B4 we simulated edema in the breast by adding a 1  cm thick, water equivalent bolus on top of the left breast (Fig. 1B). Since rotations were included in pCT, all measurements could be performed without any rotation or pitch of the phantom. Additionally, to simulate major differences between the pCT and the phantom on-couch, three spine measurements were done using CT scans from different patients with similar diameter to the phantom but a vastly different anatomy as pCT (Measurement S7-S9, Fig. 3A). In a clinical setting, the structures are evaluated on the CBCT and if necessary, manually adapted. The dose is calculated on the sCT, but shown on the CBCT. As a result, it may be hard to recognize erroneous dose calculations that arise from inaccuracies of the sCT.

### 2.4 Radiochromic film dosimetry

Treatment plans were compared to the film measurements, evaluating the delivered dose, using radiochromic film (GafChromic EBT3, Ashland Advanced Materials, Bridgewater, USA). Films were cut into 20  cm x 12.5 cm pieces for the spine and mid-plane, and 10  cm x 12.5  cm for the breast pocket (one film per pocket). On each measurement day, two calibration films were irradiated to 4  Gy dose to water (D_w_).

To assess the precision of dosimetric film measurements in the anthropomorphic phantom, measurements S0 and B0 were performed four times each. For these measurements, per film plane, we assigned three to five homogeneous regions of interest (ROI) from which the average measured dose was derived (Supplementary section D). For each location, we calculated the mean variation within the 1  cm^2^ ROI.

### 2.5 Dose calculation and film analysis

Ethos calculates dose to medium (D_m_) on a 2.5  mm resolution. The film measurements are calibrated using D_w_ and a dose resolution of 2.5  mm might result in volume averaging due to a potential dose gradient perpendicular to the film plane. Therefore, we used Eclipse to calculate D_w_ for all experiments on the sCT and pCT on a 1  mm resolution (Acuros XB, version 16.1.0). The dose planes corresponding to the film positions were exported as DICOM dose planes.

The process of film digitation and conversion to D_w,_ including the applied fit parameters, is described in Supplementary section E [31]. OmniPro I’mRT (IBA Dosimetry, Schwarzenbruck, Germany) was used to visually register the measured dose to the calculations based on the dose gradients, and to calculate gamma distribution maps [32]. We used 4  %/2 mm global dose and 10  % low dose threshold to accommodate for the measurement uncertainties that were observed in the validation measurements.

The gamma maps were cropped to avoid measurement artifacts caused by film edges, phantom edges or fixation tape used during digitation of the film. Gamma pass rate (GPR) as well as mean gamma (Gmean) are reported as a measure for correspondence between measured dose and dose calculated on pCT or sCT (GPR_pCT_, GPR_sCT_, Gmean_pCT_ and Gmean_sCT_). Additionally, GPR results for 3  %/2 mm (global dose, low dose threshold of 10  %) were calculated. An overview of the different gamma criteria used in this study is provided and motivated in Supplementary section F).

To evaluate whether the measurements show better agreement with dose calculations on pCT than on sCT we compared Gmean_pCT_ and Gmean_sCT_ using a paired Wilcoxon signed rank test (RStudio 2022.12.0) for S1-S6 and B1-B6 (Fig. 3A & 3D), p < 0.05 was considered significant.

## 3 Results

The standard deviation of mean dose in the different ROIs for the assessment of the dosimetric accuracy of the film measurements (Supplementary section D) was 1.7  % (range: − 4.1  % – 3.2  %).

**Fig. 4:**
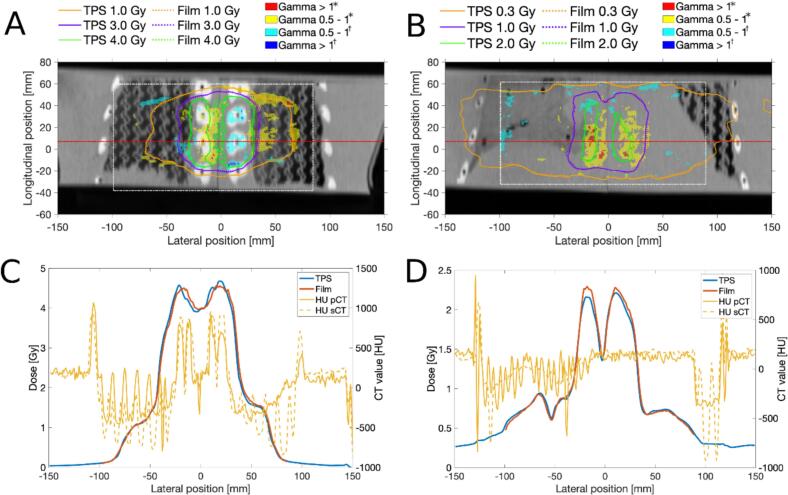
Gamma distribution and dose profiles for spine measurement S4 in plane 1 (A,C) and plane 2 (B,D). (A,B) shows the film plane with overlaid isodose lines for measured and calculated dose in addition to the gamma index. Gamma is shown in yellow/red where the measured dose exceeds the calculated (*), and in cyan/blue where it falls below (†). The white rectangles in the gamma distribution represent the selected region of interest. (C,D) Dose profile graphs are shown for the red lines in (A,B), in addition to the HU-values for the pCT and sCT. *Abbreviations: TPS = treatment planning system, pCT = planning CT, sCT = synthetic CT.* (For interpretation of the references to colour in this figure legend, the reader is referred to the web version of this article.)

**Fig. 5:**
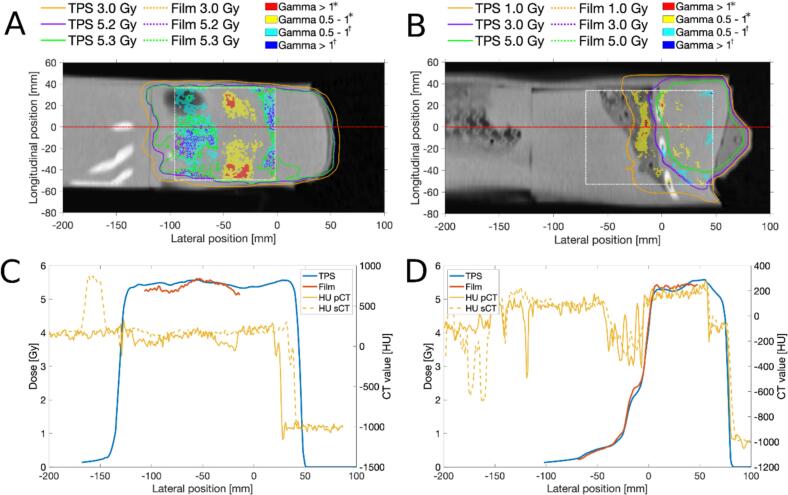
Gamma distribution and dose profiles for breast measurement B4 in plane 1 (A,C) and plane 2 (B,D). (A,B) shows the film plane with overlaid isodose lines for measured and calculated dose in addition to the gamma index. Gamma is shown in yellow/red where the measured dose exceeds the calculated (*), and in cyan/blue where it falls below (†). The white rectangles in the gamma distribution represent the selected region of interest. (C,D) Dose profile graphs are shown for the red lines in (A,B), in addition to the HU-values for the pCT and sCT. *Abbreviations: TPS = treatment planning system, pCT = planning CT, sCT = synthetic CT.* (For interpretation of the references to colour in this figure legend, the reader is referred to the web version of this article.)

Gamma pass rates (4  %/2 mm) are reported in Fig. 3. GPR as well as Gmean are reported in Table 1, results for 3  %/2 mm gamma evaluation are provided in Supplementary section H. All adaptive plans were within clinical criteria for target and OAR dose. The Wilcoxson signed rank test showed that, based on these Gmean values, the correspondence between measured dose and dose on the pCT did not significantly differ compared to the correspondence between measured dose and dose on the sCT (p = 0.15). Fig. 4, Fig. 5 show results for measurements S4 and B4 respectively, where discrepancies between calculated and measured dose of up to 0.2  Gy are visible in the dose profiles. Graphical results for all measurements are included in Supplementary section G. For all the measurements using a patients’ pCT, Gmean_pCT_ was lower than Gmean_sCT_ (p = 0.051). The differences between Gmean_pCT_ and Gmean_sCT_ were more distinct in measurement plane 2 compared to plane 1. Measurements S7-S9 showed large dose differences between the sCT and pCT (Supplementary section G, Fig. G14) for plane 2. In measurement B4, where 1  cm bolus material was added on top of the phantom, a GPR_pCT_ of 89  % was observed in measurement plane 1. The PSQA by independent dose calculation in Mobius passed our clinical criteria of >95  % pass rate for 3  %/3 mm gamma evaluation for all measured treatment plans.

**Table 1:** Mean gamma (Gmean) and gamma pass rates (GPR) for film measurements compared to dose as calculated on the planning CT and synthetic CT (pCT and sCT respectively). Gmean and GPR were calculated using 4 % / 2 mm criteria. * ID = experiment ID, SX = spine measurements, BX = breast measurements. *Abbreviations: Gmean = mean gamma, GPR = gamma pass rate, sCT = synthetic CT, pCT = planning CT.*

	**sCT**				**pCT**			
	**Plane 1**		**Plane 2**		**Plane 1**		**Plane 2**	
ID*	**Gmean**	**GPR [%]**	**Gmean**	**GPR [%]**	**Gmean**	**GPR [%]**	**Gmean**	**GPR [%]**
S1	0.39	96	0.32	99	0.34	97	0.27	100
S2	0.32	97	0.29	99	0.30	96	0.29	100
S3	0.47	96	0.23	100	0.38	99	0.25	100
S4	0.33	99	0.26	99	0.30	100	0.28	99
S5	0.33	97	0.35	95	0.32	99	0.37	94
S6	0.37	97	0.32	98	0.38	99	0.36	99
S7	0.30	97	0.35	94	0.29	98	0.30	99
S8	0.42	96	0.45	92	0.41	97	0.25	100
S9	0.37	95	0.51	86	0.29	99	0.31	99
B1	0.38	98	0.45	95	0.40	95	0.44	95
B2	0.28	100	0.31	99	0.30	99	0.22	100
B3	0.45	95	0.37	100	0.39	98	0.31	99
B4	0.47	97	0.29	100	0.47	89	0.31	100
B5	0.37	98	0.41	94	0.37	98	0.41	97
B6	0.36	98	0.24	100	0.33	98	0.31	100

## 4 Discussion

This study presented a QA method for CBCT-based oART, which is an essential step prior to clinical introduction of adaptive radiotherapy. In contrast to current literature, the test was done using a custom 3D-printed anthropomorphic thorax phantom based on an actual patient anatomy with radiographic film placed inside the phantom. Results showed the delivered dose mostly corresponds to calculated dose within clinical criteria (when including measurement uncertainties). For a few cases, the sCT did not correctly represent the actual phantom, in which the measurement was able to identify the resulting errors in dose calculation. The methodology presented in this manuscript could be used for an end-to-end test in oART in the future.

Given the observed standard deviation of 1.7  % in the validation measurements (Fig. 3), the gamma criterion of 3  % dose which we generally use in our clinic for measurements in homogeneous phantoms might be too small to detect possible problems in the workflow as it primarily shows film-measurement uncertainties. Film uncertainty varies between batches and may lead to the use of different thresholds for the gamma criterion in future measurements [32].

The spine film measurements corresponded better to the dose calculations on the pCT than on the sCT. In S6 plane 2, where the target was shifted by one vertebra, the shift of one vertebra ended up next to the diaphragm instead of next to the lungs. This difference in densities in proximity to the measurement plane caused differences in the calculated dose. Overall, considering that for most experiments the gamma pass rate was >95  %, we conclude that in most cases the sCT was an accurate representation of densities.

Another example where large differences between the pCT and CBCT led to inaccurate sCT, and therefore larger dose differences was the measurement which used a patients’ pCT as input S7-S9. Reduction of GPR_sCT_ to 86  % was caused by dose differences of maximum 7  % of prescribed dose resulting from density differences up to 1000 HU. This is like results presented in previous work, dose calculation directly on the CBCT instead of a sCT could avoid these problems [28]. The breast measurements showed less difference between dose calculations on pCT or sCT, most likely because the breast region in the phantom is less heterogeneous and local densities on the sCT are similar on the pCT. The lower GPR_pCT_ compared to GPR_sCT_ for B4, plane 1 was caused by having a bolus added to the breast during the measurement, which was not considered on the pCT. The PSQA independent dose calculation using Mobius relies on the same sCT data as the primary dose calculation in Ethos. Therefore, it is not sensitive for dosimetric errors that originate from imperfections in the sCT, as demonstrated by our experiments.

Multiple studies in current literature used phantoms for the validation of oART [21], [33], [34], [35], [36], [37], [38], [39]. Our study shows similarities with other studies on Ethos [36], [39]. However, our study showed that, when 3D printing facilities are already available, the printing of a custom 3D anthropomorphic phantom can be used as alternative to commercial phantoms, potentially lowering the costs and allowing freedom to customize the phantom for specific treatment sites and dosimetric detector types. Another study used a commercially available thorax phantom in combination with a 3D-printed breast attachment [12]. In this study, using non-adaptive volumetric modulated arc therapy with a simultaneous integrated boost to the tumor bed, a median gamma pass rate of 94.4  % was found using 3  %/2 mm gamma criteria. Our gamma criteria of 4  %/2 mm were chosen to accommodate measurement uncertainties that were observed in the validation measurements. The film measurement uncertainty was determined using four measurements per point which could be improved by increasing the number of films. Our results for 3  %/2 mm, are comparable to those reported in other research [12]. Another study focused on end-to-end testing of an adaptive breast workflow using 9-field IMRT on a commercially IMRT Thorax phantom and both ionization chamber and film measurements [39]. With gamma criteria of 3  %/3 mm, pass-rates of 90  % were found. Changes in gas volume were simulated in between treatment fractions, which led to deviations between calculated dose and dose measured with an ionization chamber. The ability to detect differences between pCT and sCT based on dose measurements is like our results for the workflow with patient scans to create a large anatomical discrepancy with the phantom.

The separate parts of the phantom create transitions of different densities within 2 mm distance to the film surface. This could have caused a high dose inhomogeneity in the direction perpendicular to the film. The accuracy of the dose calculations in presence of these in-homogeneities was limited by the voxel size of both the CT scan and the dose calculation grid. Additionally, our method was limited and did not complete a full end-to-end chain due to dose recalculation in another TPS, which might introduce minor differences with the clinical calculation. Also, the steps were carried out by physicists instead of clinical personnel, which could have resulted in a workflow which slightly differs from the clinical routine. Another limitation was the lack of geometric accuracy of the proposed method since the registration of the TPS dose plane and the film dose was done based on dose gradients instead on geometric markers. Thereby, the shape of the dose distribution was evaluated rather than the absolute position of the dose distribution. Another limitation of this phantom was the inability to deform or change the anatomy before or during treatment. As a result, we depended on changes in the positioning of the phantom and changes in target contours instead of changes in the actual anatomy. In clinical practice not only the positioning of the patient might change, but in most online adaptive treatment sites also anatomical changes may occur which can impact the treatment plan optimization. Therefore, when performing end-to-end testing or QA on inter-fraction dose accumulation, a deformable phantom would be preferred. We aimed to simulate this by using patient scans in treatment planning with a different anatomy compared to the phantom. For follow-up on our study, we provide a number of practical recommendations. Firstly, to mitigate steep dose gradients in the proximity of the film, the dose grid used for calculation should be as small as possible (1  mm in our case). Secondly, if the film is calibrated to Dw, also calculate the delivered dose in Dw [40]. Thirdly, deformation could be mimicked by digitally deforming the phantom CT. Fourthly, a next step in this method would be to complete the end-to-end cycle by completing a complete patient cycle, including clinical tasks for the radiation oncologist and radiation therapists. Finally, we recommend repeating the film validation process when carrying out the measurements using a new film batch.

In summary, we have developed a QA method for a CBCT-based online adaptive radiotherapy workflow, based on film measurements in a 3D-printed anthropomorphic phantom. We were able to identify dosimetric errors resulting from imperfections of the sCT. The clinical workflow for irradiation of spine and breast tumors was verified and proved that to be safe and robust despite imperfections that can occur in the sCT.

## Funding

Amsterdam UMC received a grant from Varian Medical Systems for the current work.

## Declaration of competing interest

The authors declare the following financial interests/personal relationships which may be considered as potential competing interests: Wilko Verbakel is employed by both Amsterdam UMC and Varian Medical Systems, and has received travel honoraria from Varian Medical Systems. Eva Versteijne, Koen Nelissen and Daan Hoffmans have no disclosures.
