# Supplementary material for: Quality assurance of online adaptive radiotherapy workflows using film dosimetry in a 3D printed thorax anthropomorphic phantom
Source: Phys Imaging Radiat Oncol. 2026 Jan 22;37:100909. doi: 10.1016/j.phro.2026.100909 (PMC12873733; doi:10.1016/j.phro.2026.100909)

**Supplementary Materials**

Additional info for the manuscript titled: “End-to-end quality assurance of online adaptive radiotherapy workflows using film dosimetry in a 3D printed anthropomorphic phantom.”

By: Daan Hoffmans and Koen Nelissen, Amsterdam UMC

**Supplementary section A: Phantom construction**

A detailed description of the production process of the phantom is described elsewhere:

Hazelaar C, van Eijnatten M, Dahele M, Wolff J, Forouzanfar T, Slotman B, et al. Using 3D printing techniques to create an anthropomorphic thorax phantom for medical imaging purposes. Med Phys 2018;45:92–100. <https://doi.org/10.1002/mp.12644>

Table A1 and Figure A1 provide further details.

**Table A1:** Image segmentation characteristics for human tissue and corresponding phantom materials

| **Tissue** | **Phantom material** | **Printer** | **Production consumable** | **Patient HU**  **Mean (range)** | **Phantom HU**  **Mean (range)** |
| --- | --- | --- | --- | --- | --- |
| Soft Tissue | Silicone |  | Dragon Skin® 30, (Smooth-On, Macungie, PA, USA) | -43 (-117 to 285) | 184 (95 to 274) |
| Bone | Plaster | Zcorp 650® (3D Systems, Rock Hill, SC, USA) | ZCorp® zp151 high-performance powder | 371 (150 to 1437) | 852 (544 to 1238) |
| Lungs | Polylactic acid (PLA) |  |  | -378 (-605 to -97) | -524 (-1180 to 371) |
| Outer Shell | Nylon | Selective Laser Sintering (SLS), (EOS GmbH, Krailling, Germany) | EOS PA2200 | Not applicable | -19 (-101 to 114) |
| Sensor Guides | PolyEthylene infusion line |  |  | Not applicable | Not applicable |


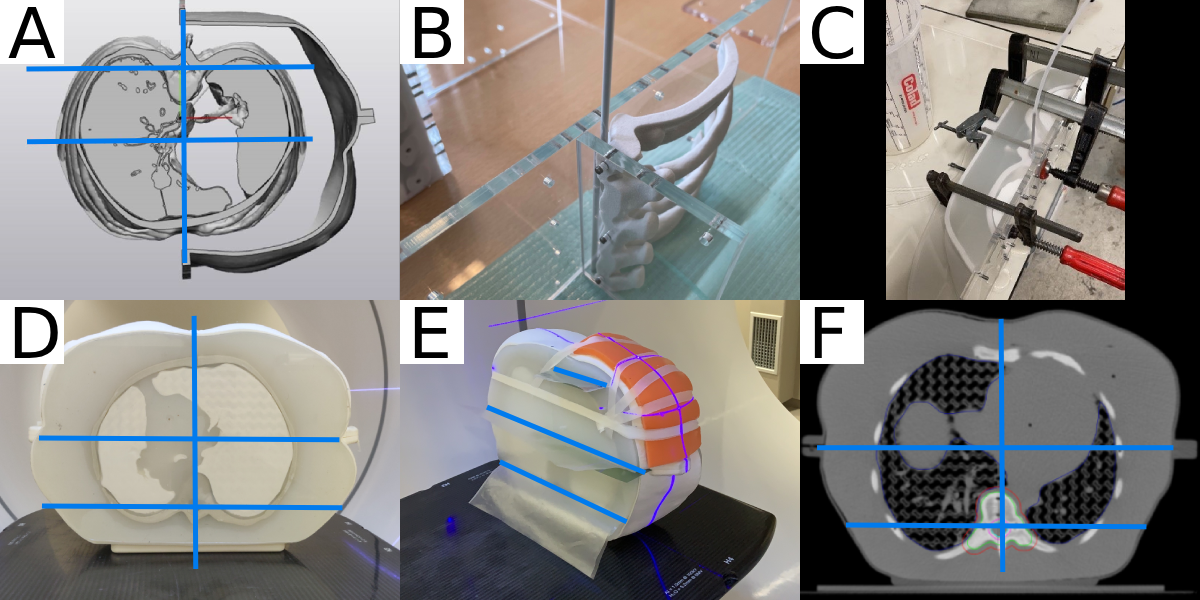
**Figure A1:** Phantom production images are shown in subfigures A-C: A represents the digital model used for printing, B shows the printed bone structure of one part, and C shows part of the phantom being cast using silicone.

**Supplementary section B: Treatment planning templates in Ethos**


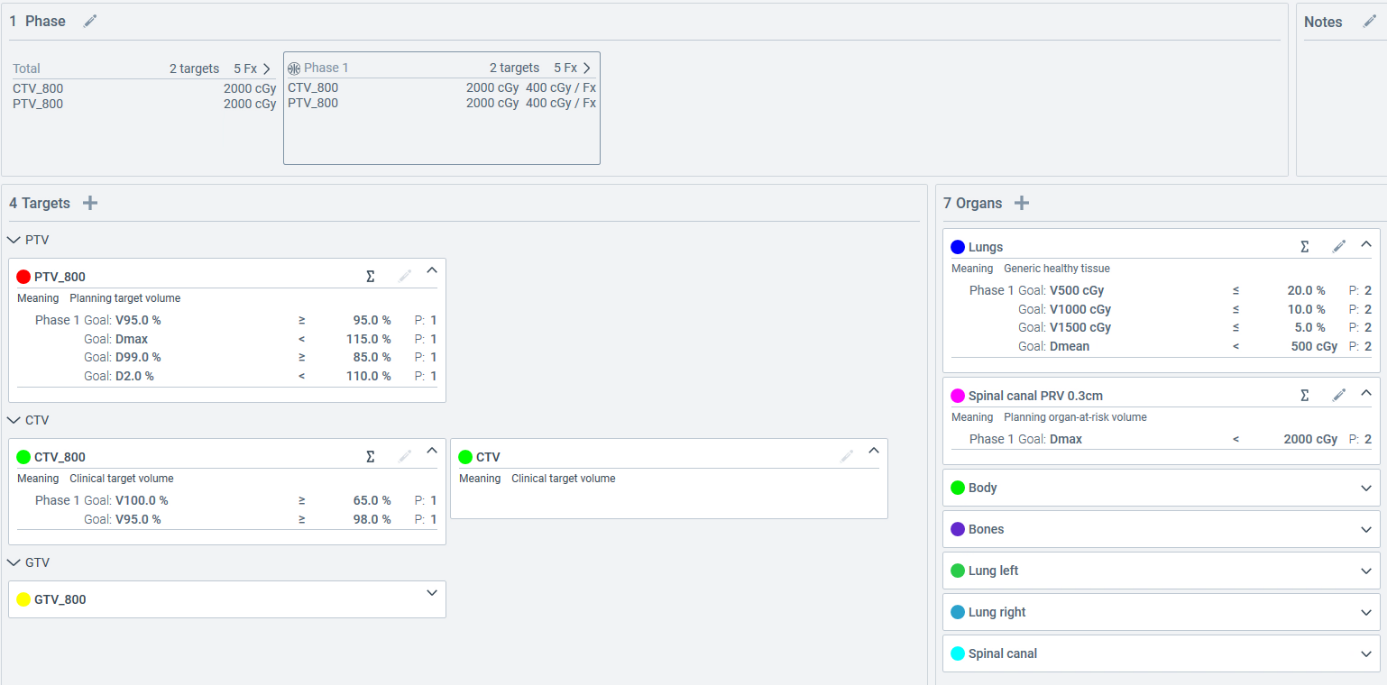
**Figure B1:** Clinical goals in Ethos system for spine measurements.


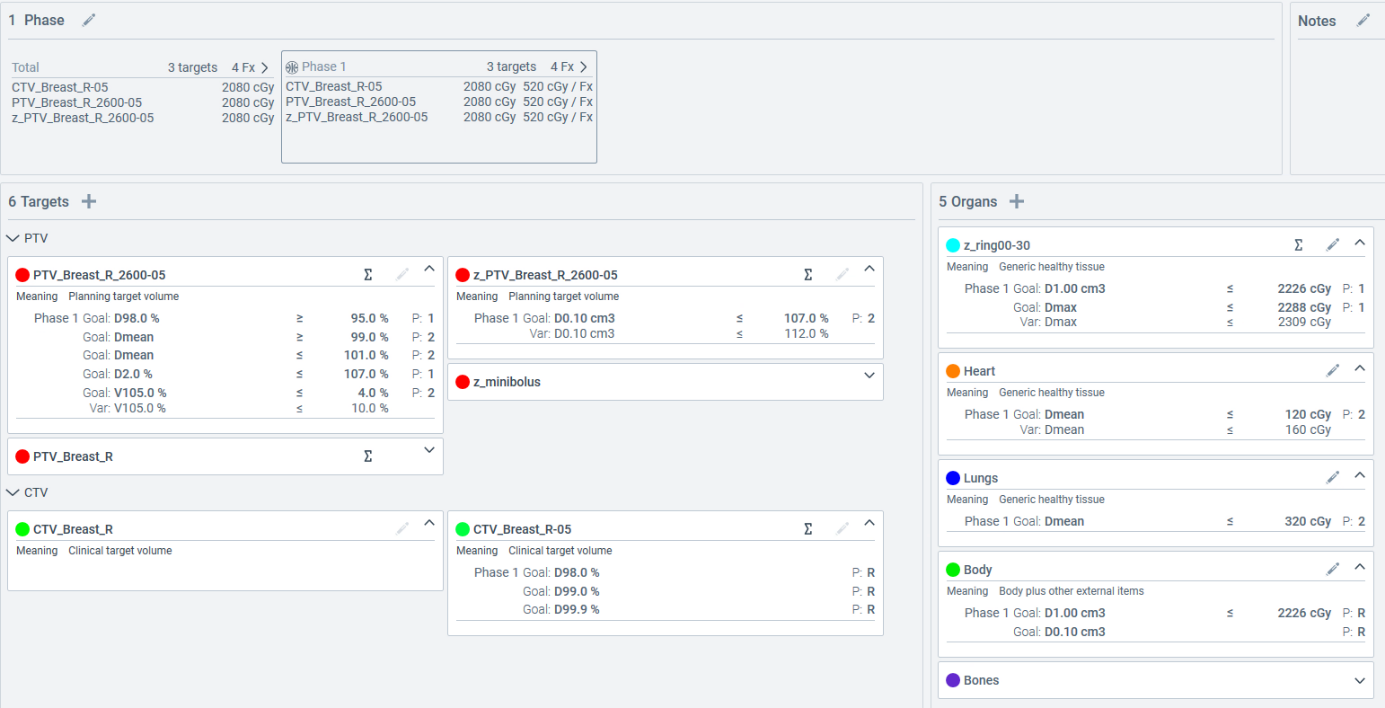
**Figure B2:** Clinical goals in Ethos system for breast measurements.

**Supplementary section C: Overview of performed measurements**

**Table C1:** Overview of spine measurements performed, results are shown in the main text Figure 2.

| **Measurement ID** | **CT used for treatment planning** | **Variation between planning CT and on-couch CBCT** | **Additional information** |
| --- | --- | --- | --- |
| S0 | CT_standard | No variation | Four validation measurements were done with this plan to evaluate film accuracy. |
| S1 | CT_standard | No variation |  |
| S2 | CT_pitch | ~6 degree pitch |  |
| S3 | CT_pitch_rotate | ~6 degree pitch and ~4 degree rotate |  |
| S4 | CT_standard | CTV expansion 1 | CTV was expanded on-couch to include another vertebra |
| S5 | CT_standard | CTV expansion 2 | CTV was expanded on-couch to include soft tissue around the vertebra |
| S6 | CT_standard | CTV moved | CTV was moved on-couch to another vertebra |
| S7 | CT_patient_1 | Completely different anatomy, patient CT used compared to phantom anatomy on-couch |  |
| S8 | CT_patient_2 |  |  |
| S9 | CT_patient_3 |  |  |

**Table C2:** Overview of breast measurements performed, results are shown in the main text Figure 3.

| **Measurement ID** | **CT used for treatment planning** | **Variation between planning CT and on-couch CBCT** | **Additional information** |
| --- | --- | --- | --- |
| B0 | CT_standard | No variation | Four validation measurements were done with this plan to evaluate film accuracy. |
| B1 | CT_standard | No variation |  |
| B2 | CT_pitch | ~6 degree pitch |  |
| B3 | CT_pitch_rotate | ~6 degree pitch and ~4 degree rotate |  |
| B4 | CT_standard | Change in body | A bolus was placed on the phantom at the breast target |
| B5 | CT_standard | CTV expansion | CTV was expanded on-couch to include more soft tissue around the breast target |
| B6 | CT_standard | CTV cropped | CTV was cropped to only include a partial breast |

**Supplementary section D: Regions of Interest for evaluation of local film variation**


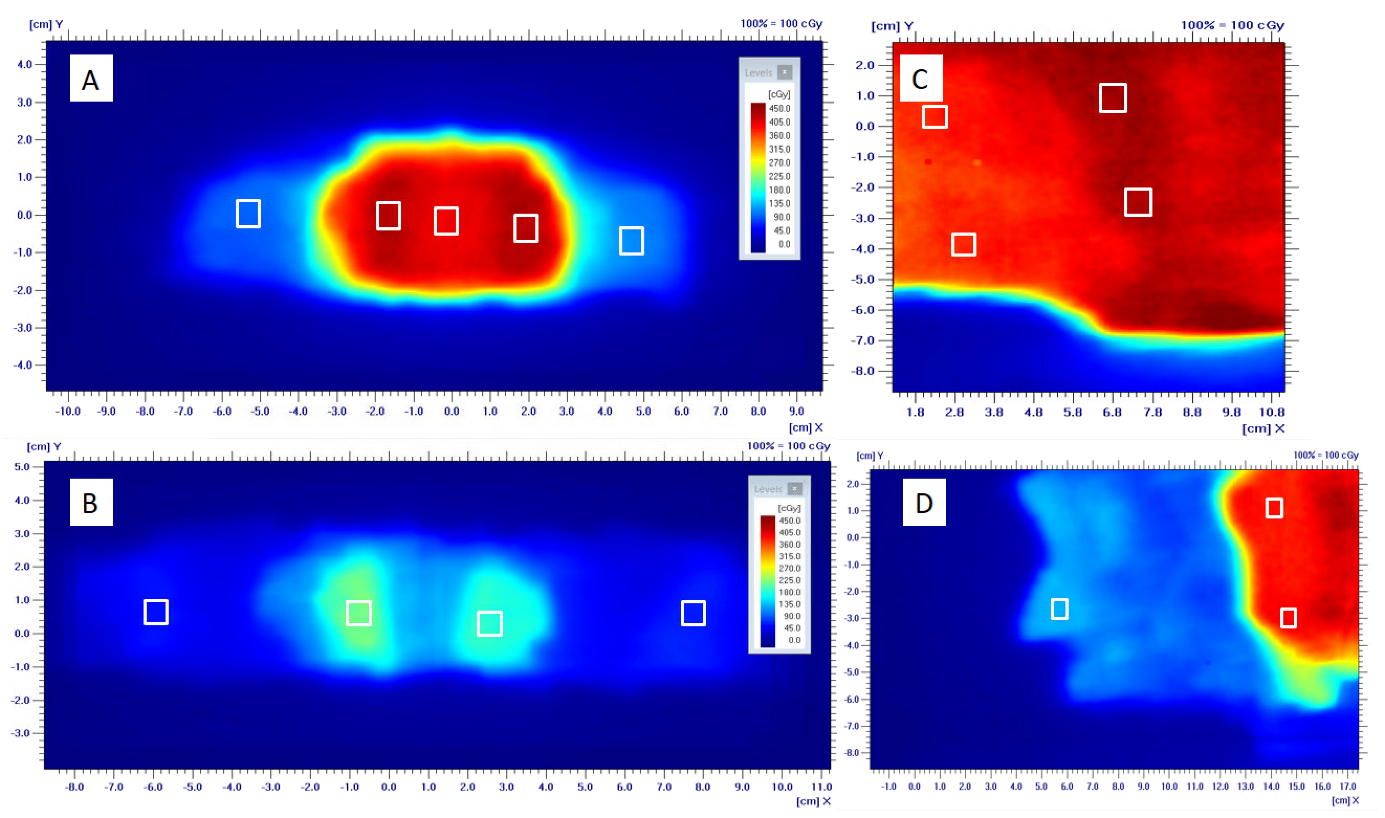
**Figure D1:** ROI’s used for assessment of local variation in film sensitivity. A & B: Spine plane 1 and 2 respectively. C & D: Breast plane 1 and 2 respectively.

**Supplementary section E: Film calibration**

Radiochromic films were digitized using a commercial flatbed scanner (*Epson Expression 12000XL, Suwa, Japan*) in transmission mode, resolution of 72 dots per inch, 16 bit per color channel. Also, a background scan of the empty scanner bed was acquired *(I_0_*)*.* Then, in-house developed software (Matlab 2018a, The Mathworks, Natick, USA) was used to correct for the lateral scan artefact (LRA), to convert to dose and to correct for daily output variations of the treatment machine using the daily calibration films.

All films, (both plan measurements as well as the daily calibration films) were processed using the following procedure:

The scanner red channel signal *(I_t,red_*) was converted to optical density (OD_red_) according to:

$$OD_{red} = \log10\left( \frac{I_{0,red}}{I_{t,red}} \right)$$

Optical Density was corrected for LRA using a polynomial surface fit (3^rd^ order lateral position (L, pixels), 2^nd^ order OD):

$$OD_{red,LRA} = p_{00}+p_{10}L+p_{01}OD+p_{20}L^{2}+p_{11}L\cdot OD+p_{02}OD^{2}+p_{30}L^{3}+p_{21}L^{2}\cdot OD+p_{12}L\cdot OD^{2}$$

The fit parameters *p_xy_* were established earlier for this combination of film type and flatbed scanner, and are provided in Table S4.

**Table E1** Fit parameters for correction of the lateral scan artefact in the red color channel.

| Parameter | Value |
| --- | --- |
| p_00_ | 0.9843 |
| p_10_ | -2.085e-5 |
| p_01_ | 4.013e-2 |
| p_20_ | 1.406e-7 |
| p_11_ | -5.275e-5 |
| p_02_ | -2.303e-2 |
| p_30_ | 2.173e-10 |
| p_21_ | 5.075e-7 |
| p_12_ | 1.694e-5 |
| R^2^ | 0.981 |

Then, LRA-corrected OD was converted to dose using a rational function:

$$D_{red} = \frac{x_{1}OD_{red,LRA}+x_{2}}{x_{3}OD_{red,LRA}+x_{4}OD_{red,LRA}+x_{5}}$$

The fit parameters *x_1_-x_5_* were determined earlier during commissioning of the film batch.

**Table E2** Fit parameters for conversion of Optical Density to dose.

| Parameter | Value |
| --- | --- |
| x_1_ | 1.25e-2 |
| x_2_ | 1 |
| x_3_ | 1.8287e-5 |
| x_4_ | -2.6048 |
| x_5_ | 6.7501e4 |
| R^2^ | 0.999 |

Finally, the dose of plan measurements (D_plan_) was corrected for daily output variations of the treatment machine using the daily calibration film (D_cal_) which were irradiated to a known dose (D_ref_):

$$D_{plan,cal}=D_{plan}\cdot\left( \frac{D_{ref}}{D_{cal}} \right)$$

**Supplementary section F: Gamma analysis**

In this study, three different gamma criteria were used for comparison of dose distributions. An overview is provided in Table S6.

**Table F1** Gamma criteria used for datasets. TPS: Treatment Planning System.

| Origin dataset 1 | Origin dataset 2 | Global dose difference [%] | Distance to agreement [mm] | Low dose threshold [%] |
| --- | --- | --- | --- | --- |
| TPS | Mobius | 3 | 3 | 10 |
| TPS | Film | 4 | 2 | 10 |
| TPS | Film | 3 | 2 | 10 |

The gamma criteria for Mobius that are presented in this study are according to clinical settings that we used at the time of the measurements. The distance to agreement here is set to 3 mm to accommodate for the dose resolution of Mobius which is 2.5 mm.

For the comparison of film measurements, we use a distance to agreement of 2 mm, which is feasible because of the high spatial resolution of the film measurements. In the body of the article, we use a dose criterion of 4 %, based on the reproducibility that we observed in the verification measurements. However, in order to be able to compare our results to literature we also provide values for 3 % / 2 mm in the supplementary materials.

**Supplementary section G: Results from individual measurements (gamma, HU and sCT)**

Discussion on film uncertainty measurements:

The validation measurements do have some outliers in the gamma pass rate, this is due to the inherent uncertainty of film dosimetry.


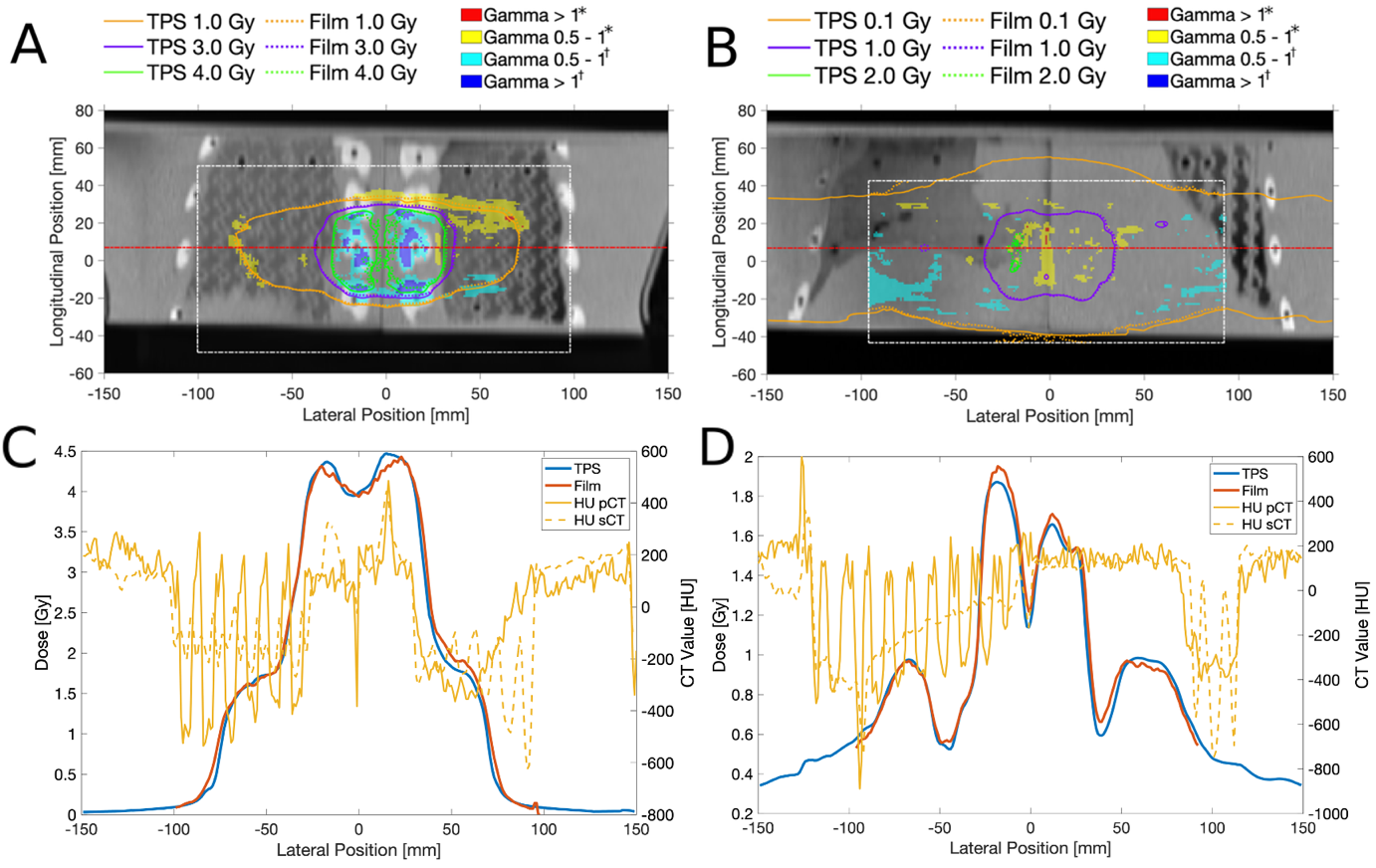


**Figure G1** Gamma distribution and dose profiles for spine measurement S1 in plane 1 (A ,C) and plane 2 (B,D). (A,B) shows the film plane with overlayed isodose lines for measured and calculated dose in addition to the gamma index. Gamma is shown in yellow/red where the measured dose exceeds the calculated, and in cyan/blue where it falls below. The white rectangles in the gamma distribution represent the selected region of interest. (C,D) Dose profile graphs are shown for the red lines in (A,B), in addition to the HU-values for the pCT and sCT.


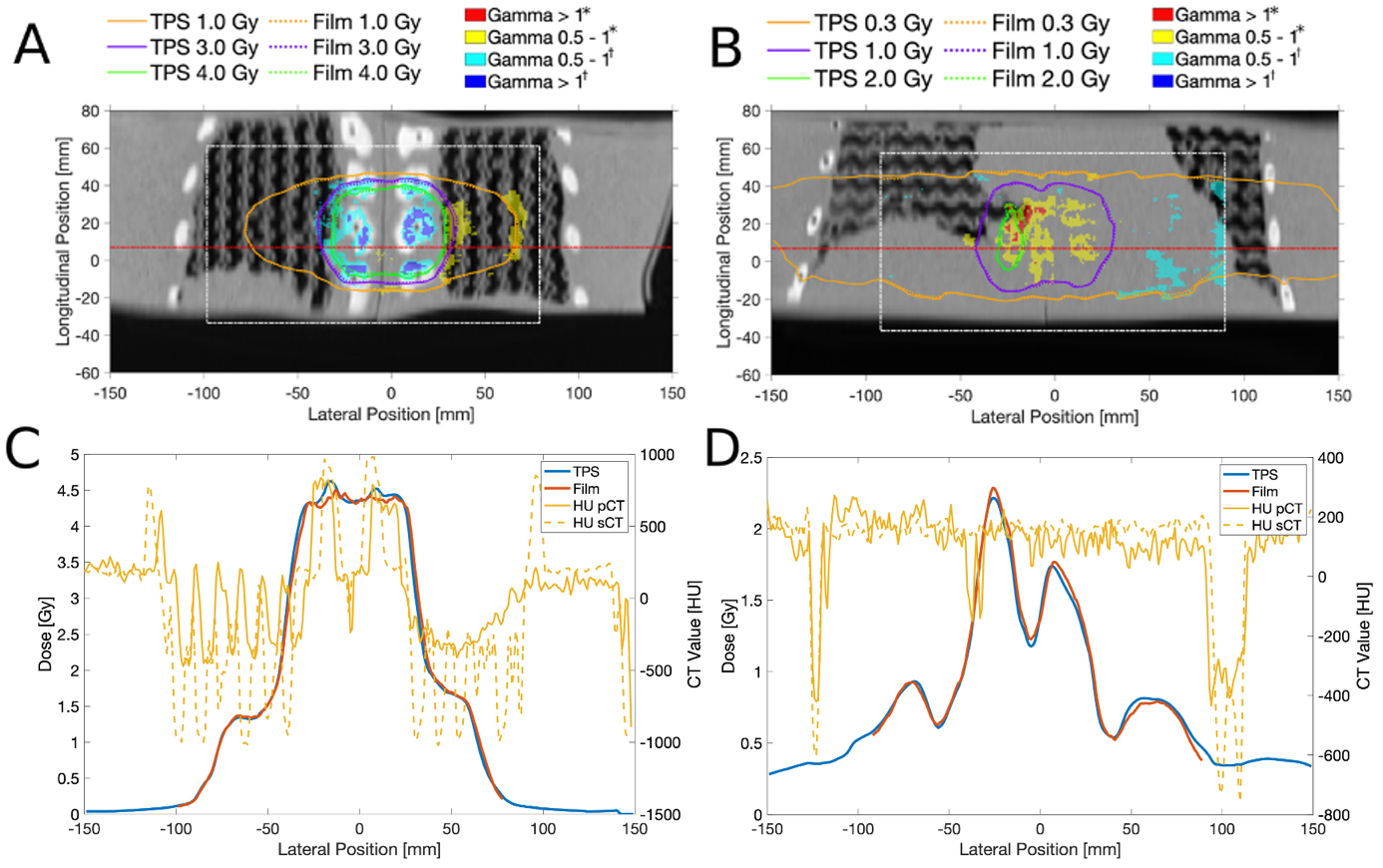
**Figure G2:** Gamma distribution and dose profiles for spine measurement S2 in plane 1 (A ,C) and plane 2 (B,D). (A,B) shows the film plane with overlayed isodose lines for measured and calculated dose in addition to the gamma index. Gamma is shown in yellow/red where the measured dose exceeds the calculated, and in cyan/blue where it falls below. The white rectangles in the gamma distribution represent the selected region of interest. (C,D) Dose profile graphs are shown for the red lines in (A,B), in addition to the HU-values for the pCT and sCT.


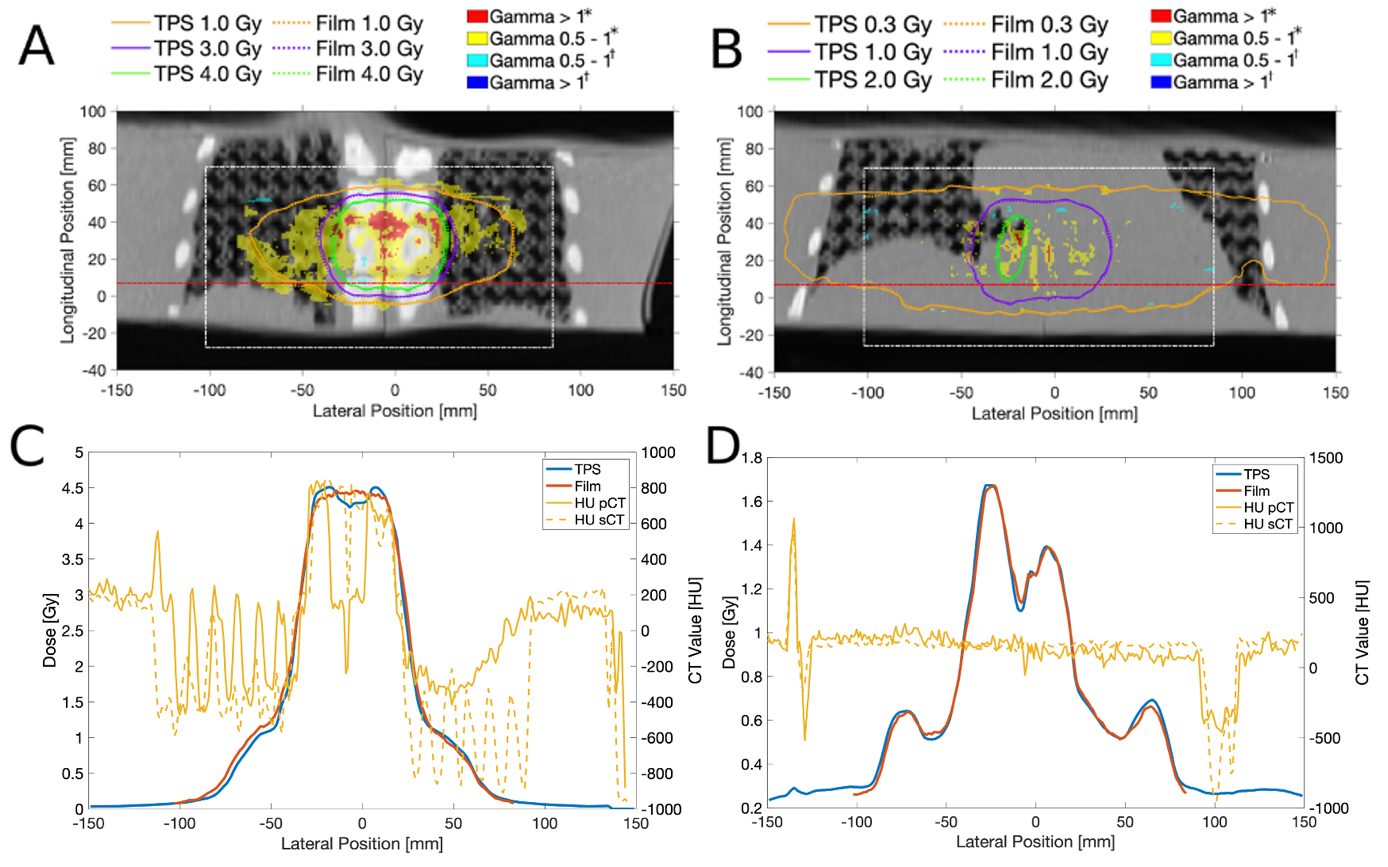
**Figure G3:** Gamma distribution and dose profiles for spine measurement S3 in plane 1 (A ,C) and plane 2 (B,D). (A,B) shows the film plane with overlayed isodose lines for measured and calculated dose in addition to the gamma index. Gamma is shown in yellow/red where the measured dose exceeds the calculated, and in cyan/blue where it falls below. The white rectangles in the gamma distribution represent the selected region of interest. (C,D) Dose profile graphs are shown for the red lines in (A,B), in addition to the HU-values for the pCT and sCT.


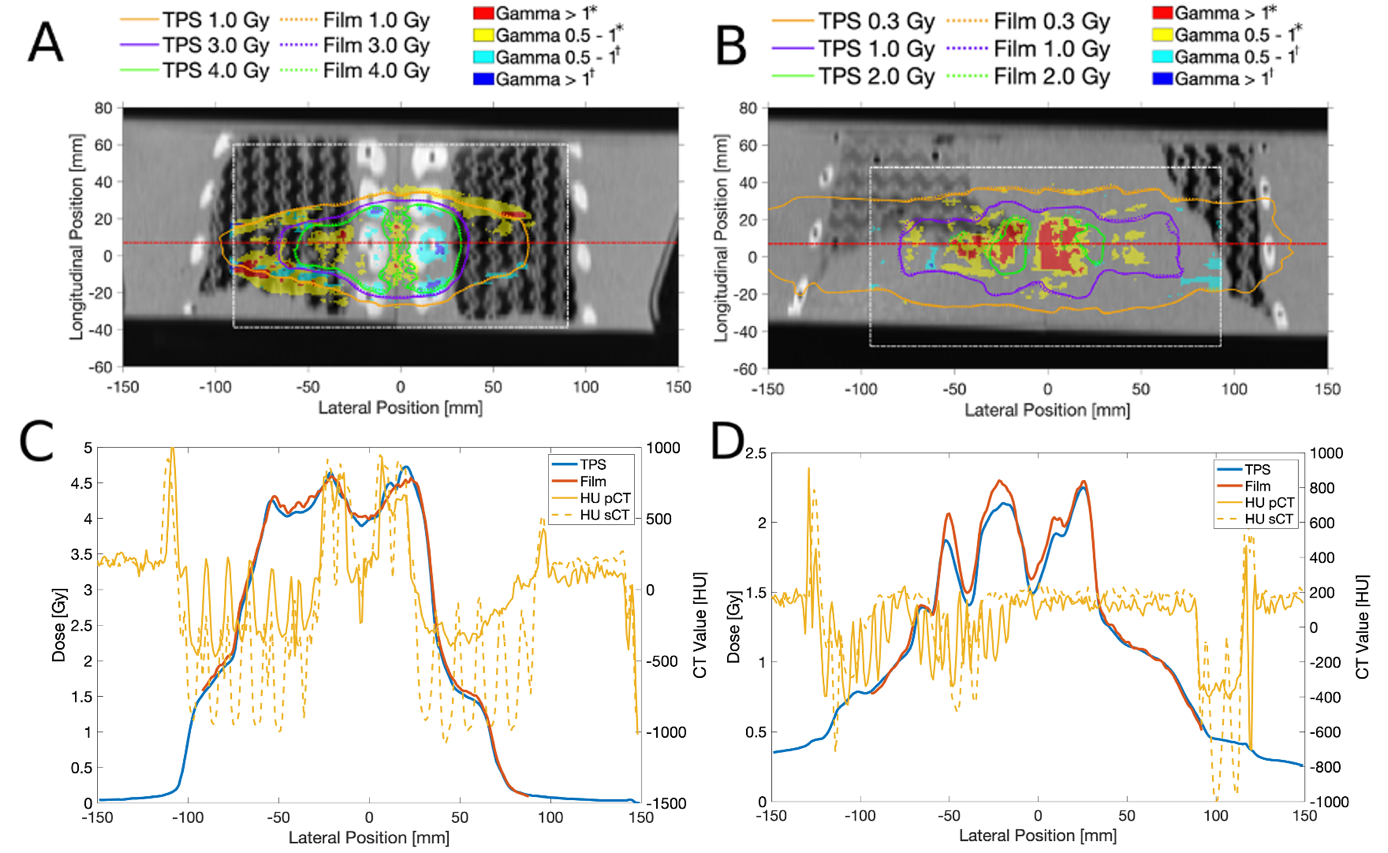


**Figure G4:** Gamma distribution and dose profiles for spine measurement S5 in plane 1 (A ,C) and plane 2 (B,D). (A,B) shows the film plane with overlayed isodose lines for measured and calculated dose in addition to the gamma index. Gamma is shown in yellow/red where the measured dose exceeds the calculated, and in cyan/blue where it falls below. The white rectangles in the gamma distribution represent the selected region of interest. (C,D) Dose profile graphs are shown for the red lines in (A,B), in addition to the HU-values for the pCT and sCT.


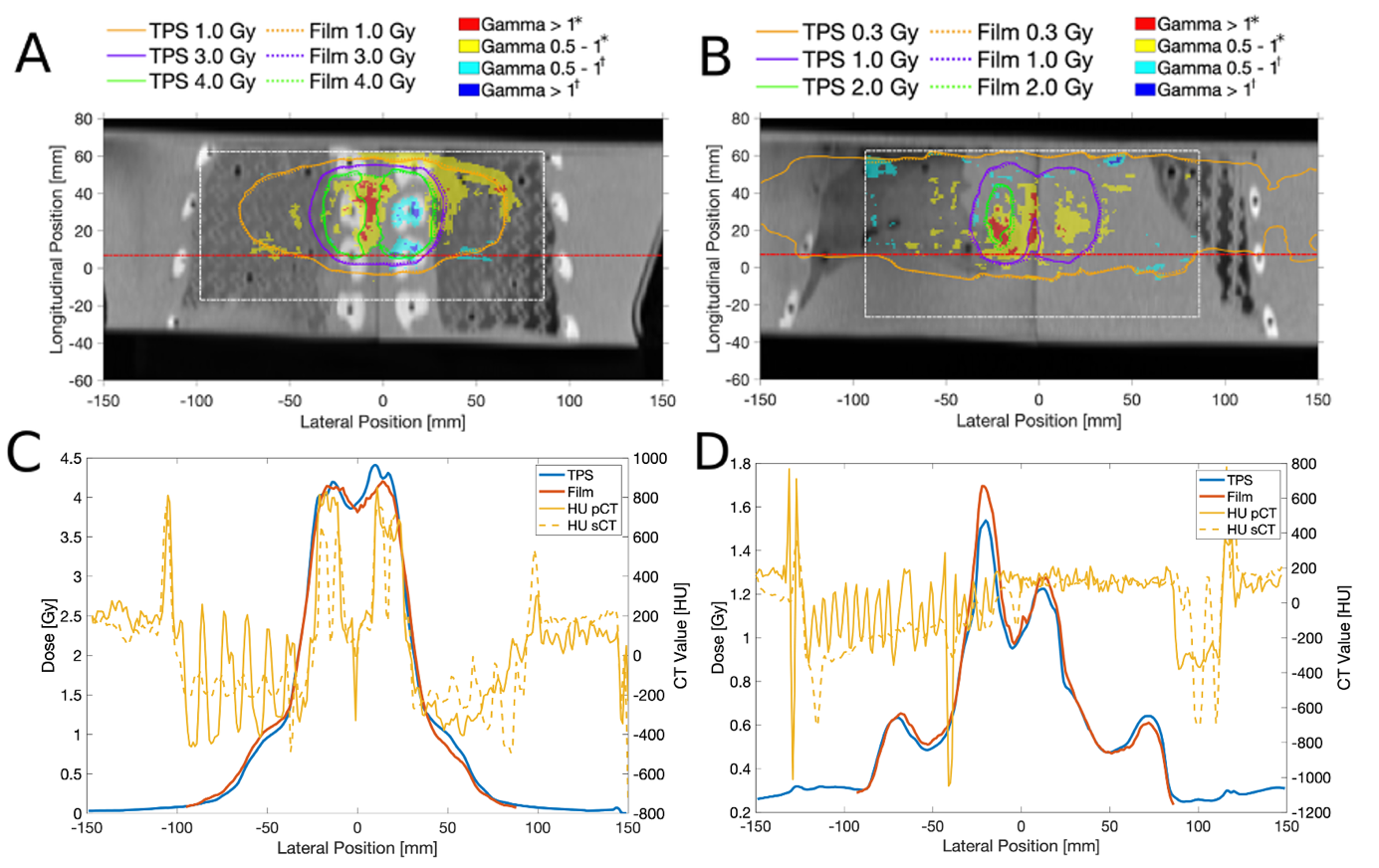
**Figure G5:** Gamma distribution and dose profiles for spine measurement S6 in plane 1 (A ,C) and plane 2 (B,D). (A,B) shows the film plane with overlayed isodose lines for measured and calculated dose in addition to the gamma index. Gamma is shown in yellow/red where the measured dose exceeds the calculated, and in cyan/blue where it falls below. The white rectangles in the gamma distribution represent the selected region of interest. (C,D) Dose profile graphs are shown for the red lines in (A,B), in addition to the HU-values for the pCT and sCT.


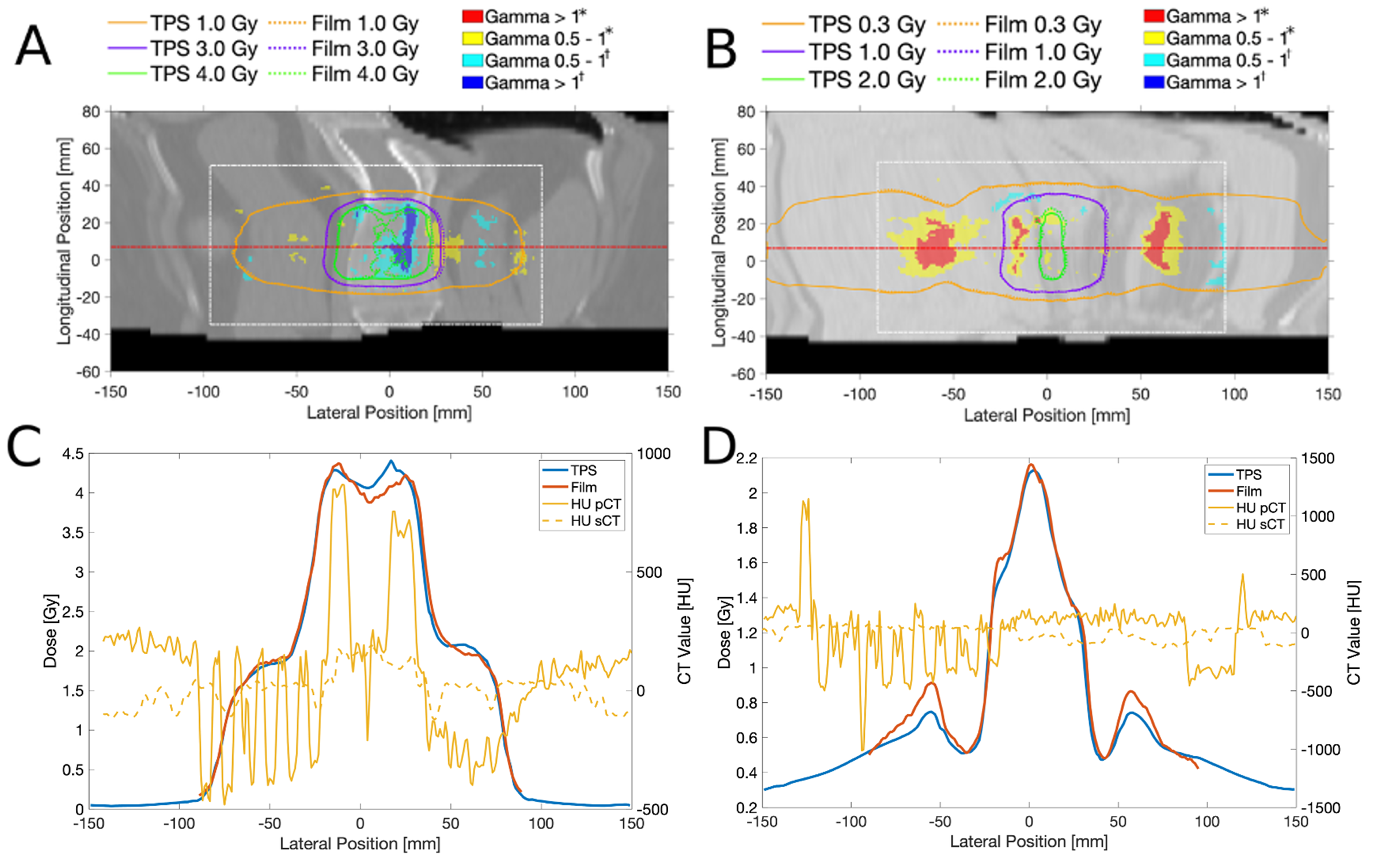
**Figure G6:** Gamma distribution and dose profiles for spine measurement S7 in plane 1 (A ,C) and plane 2 (B,D). (A,B) shows the film plane with overlayed isodose lines for measured and calculated dose in addition to the gamma index. Gamma is shown in yellow/red where the measured dose exceeds the calculated, and in cyan/blue where it falls below. The white rectangles in the gamma distribution represent the selected region of interest. (C,D) Dose profile graphs are shown for the red lines in (A,B), in addition to the HU-values for the pCT and sCT.


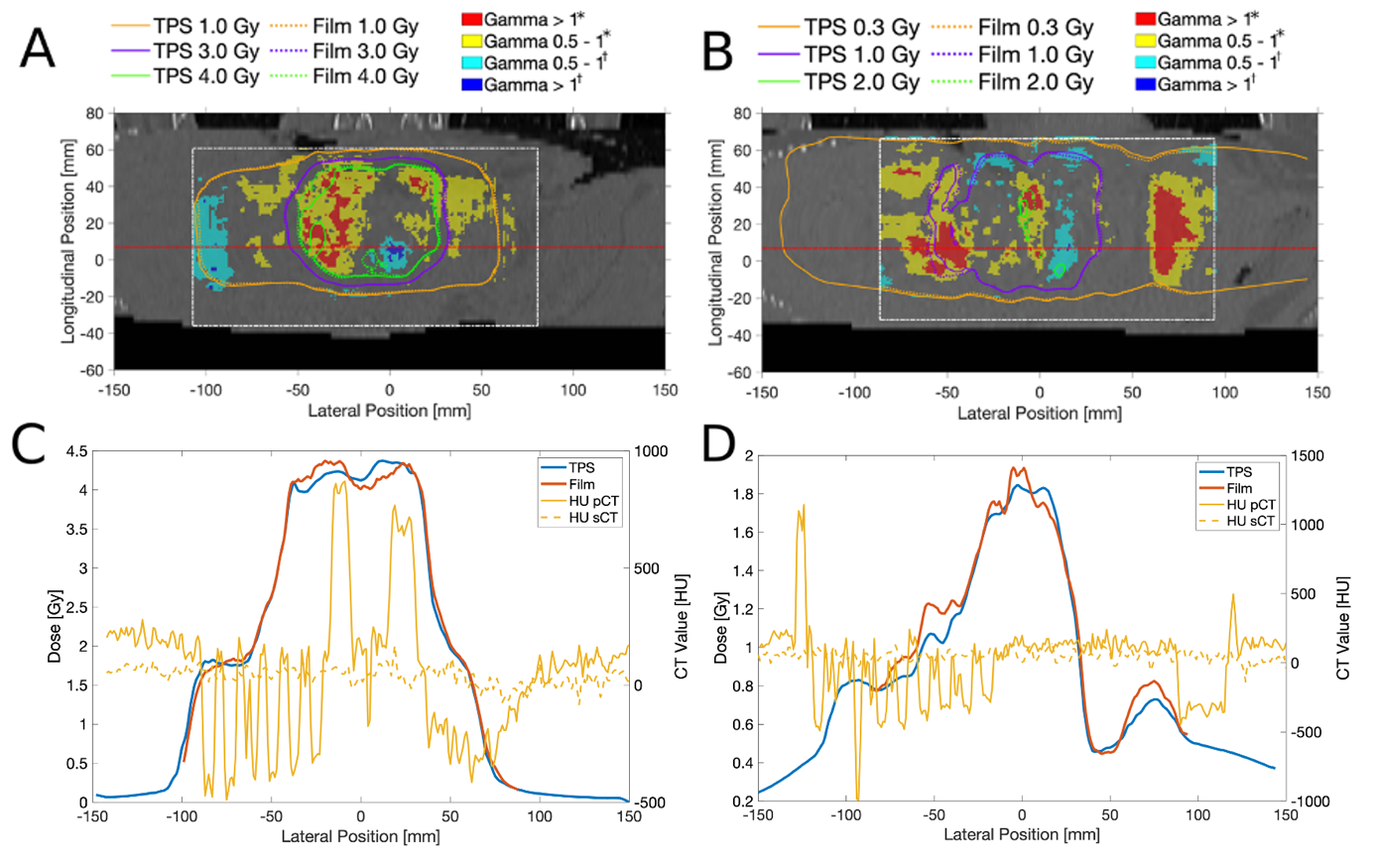
**Figure G7:** Gamma distribution and dose profiles for spine measurement S8 in plane 1 (A ,C) and plane 2 (B,D). (A,B) shows the film plane with overlayed isodose lines for measured and calculated dose in addition to the gamma index. Gamma is shown in yellow/red where the measured dose exceeds the calculated, and in cyan/blue where it falls below. The white rectangles in the gamma distribution represent the selected region of interest. (C,D) Dose profile graphs are shown for the red lines in (A,B), in addition to the HU-values for the pCT and sCT.


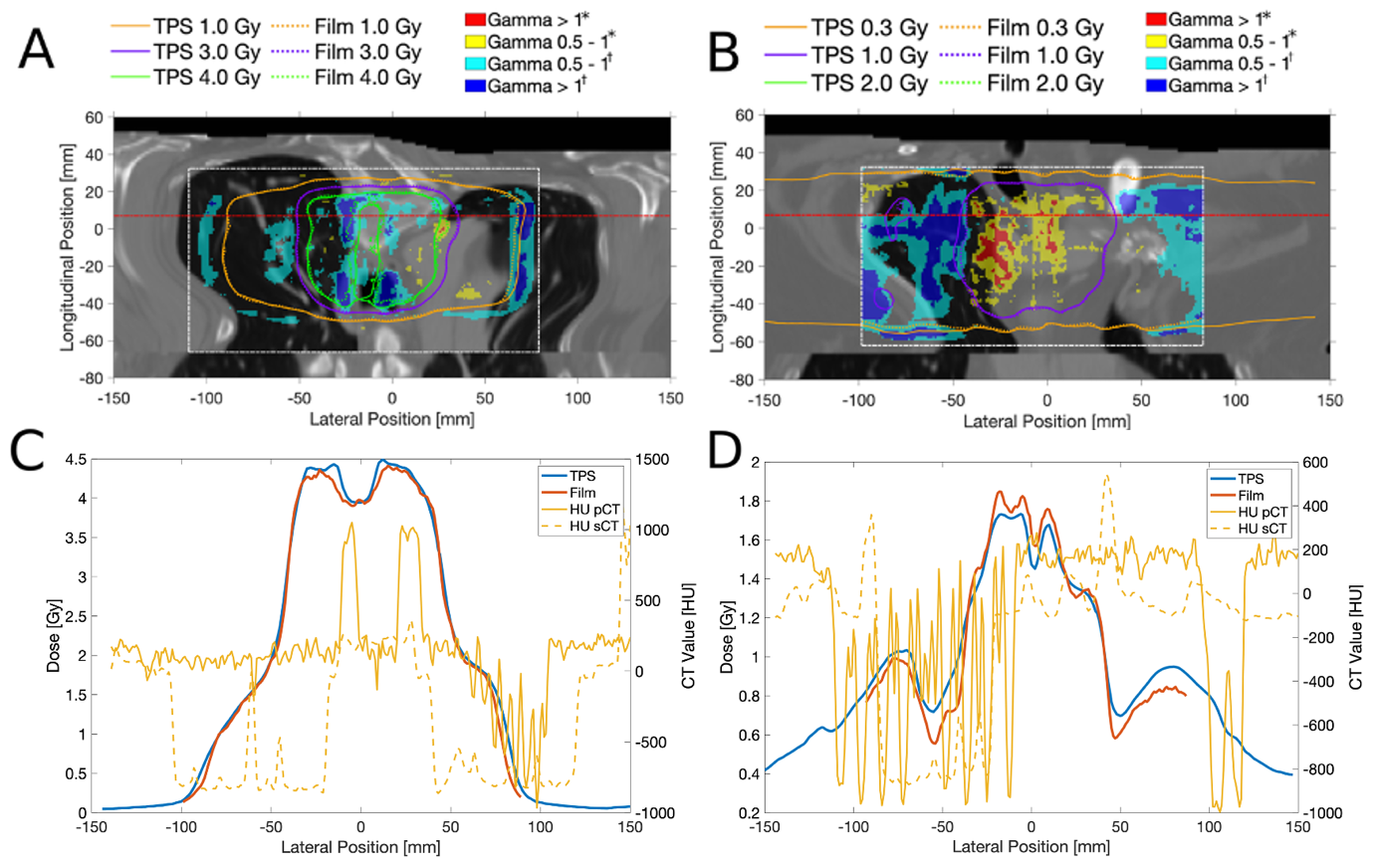
**Figure G8:** Gamma distribution and dose profiles for spine measurement S9 in plane 1 (A ,C) and plane 2 (B,D). (A,B) shows the film plane with overlayed isodose lines for measured and calculated dose in addition to the gamma index. Gamma is shown in yellow/red where the measured dose exceeds the calculated, and in cyan/blue where it falls below. The white rectangles in the gamma distribution represent the selected region of interest. (C,D) Dose profile graphs are shown for the red lines in (A,B), in addition to the HU-values for the pCT and sCT.


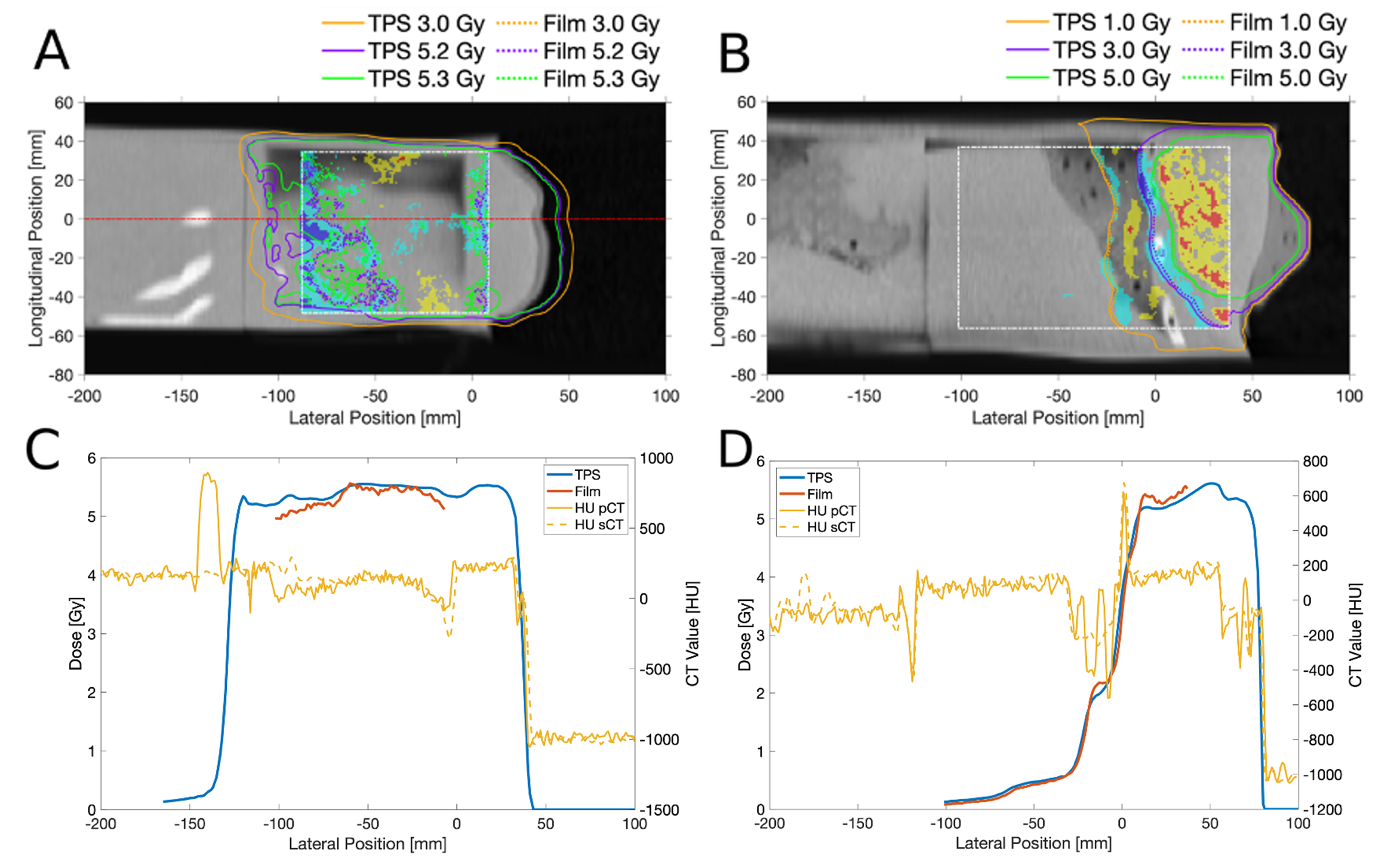


**FigureG9:** Gamma distribution and dose profiles for breast measurement B1 in plane 1 (A ,C) and plane 2 (B,D). (A,B) shows the film plane with overlayed isodose lines for measured and calculated dose in addition to the gamma index. Gamma is shown in yellow/red where the measured dose exceeds the calculated, and in cyan/blue where it falls below. The white rectangles in the gamma distribution represent the selected region of interest. (C,D) Dose profile graphs are shown for the red lines in (A,B), in addition to the HU-values for the pCT and sCT.


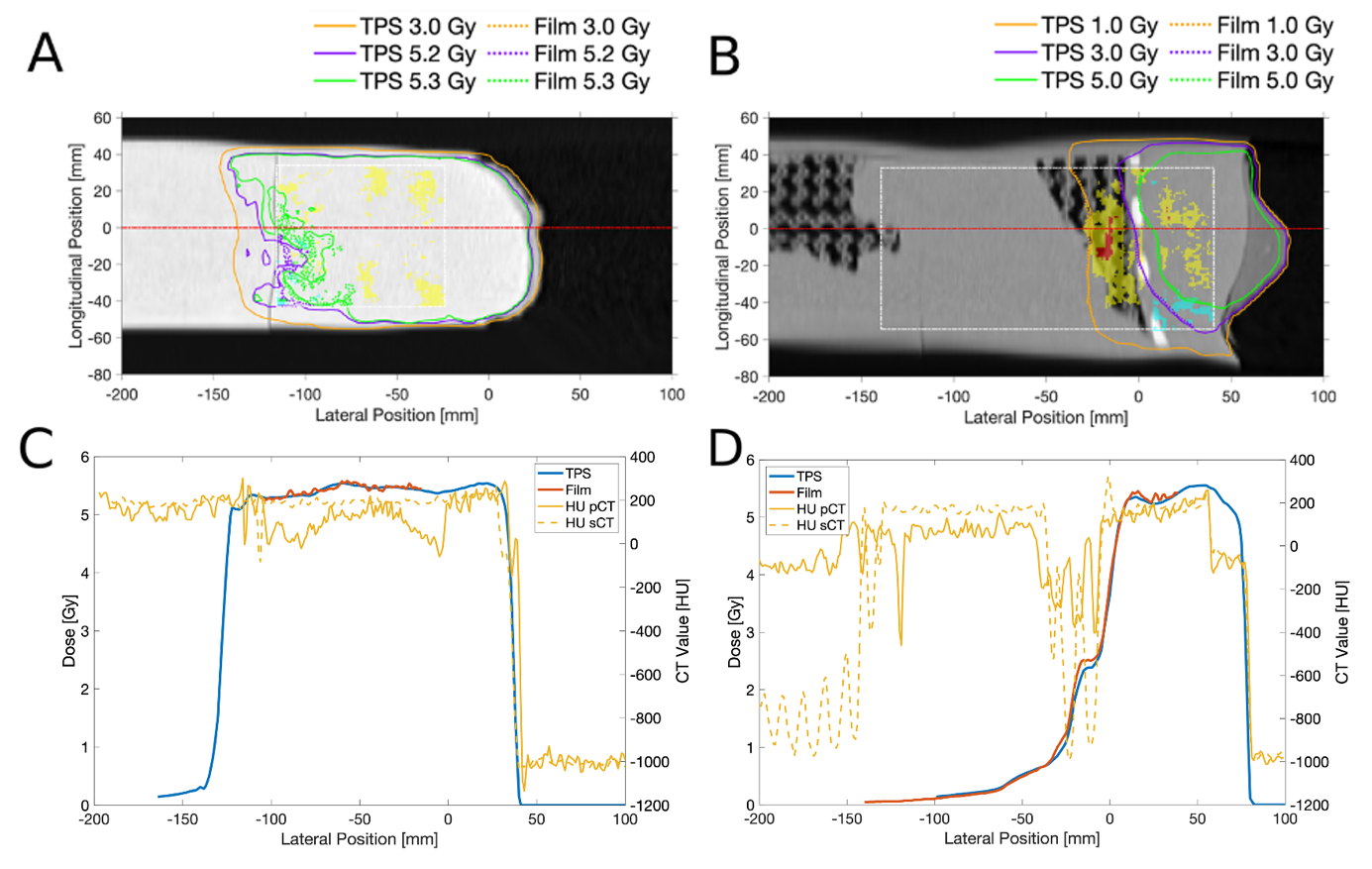


**Figure G10:** Gamma distribution and dose profiles for breast measurement B2 in plane 1 (A ,C) and plane 2 (B,D). (A,B) shows the film plane with overlayed isodose lines for measured and calculated dose in addition to the gamma index. Gamma is shown in yellow/red where the measured dose exceeds the calculated, and in cyan/blue where it falls below. The white rectangles in the gamma distribution represent the selected region of interest. (C,D) Dose profile graphs are shown for the red lines in (A,B), in addition to the HU-values for the pCT and sCT.


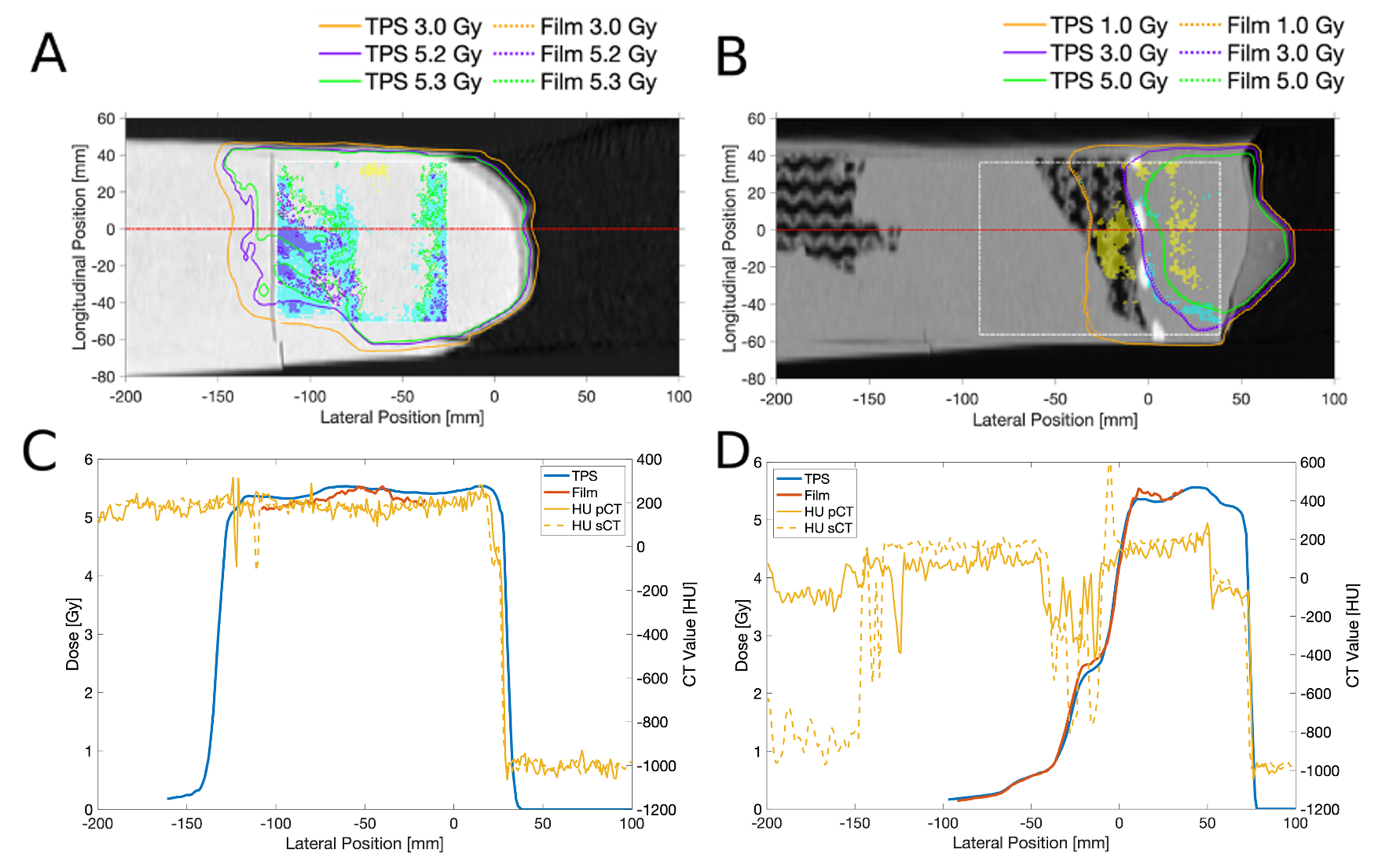


**Figure G11:** Gamma distribution and dose profiles for breast measurement B3 in plane 1 (A ,C) and plane 2 (B,D). (A,B) shows the film plane with overlayed isodose lines for measured and calculated dose in addition to the gamma index. Gamma is shown in yellow/red where the measured dose exceeds the calculated, and in cyan/blue where it falls below. The white rectangles in the gamma distribution represent the selected region of interest. (C,D) Dose profile graphs are shown for the red lines in (A,B), in addition to the HU-values for the pCT and sCT.


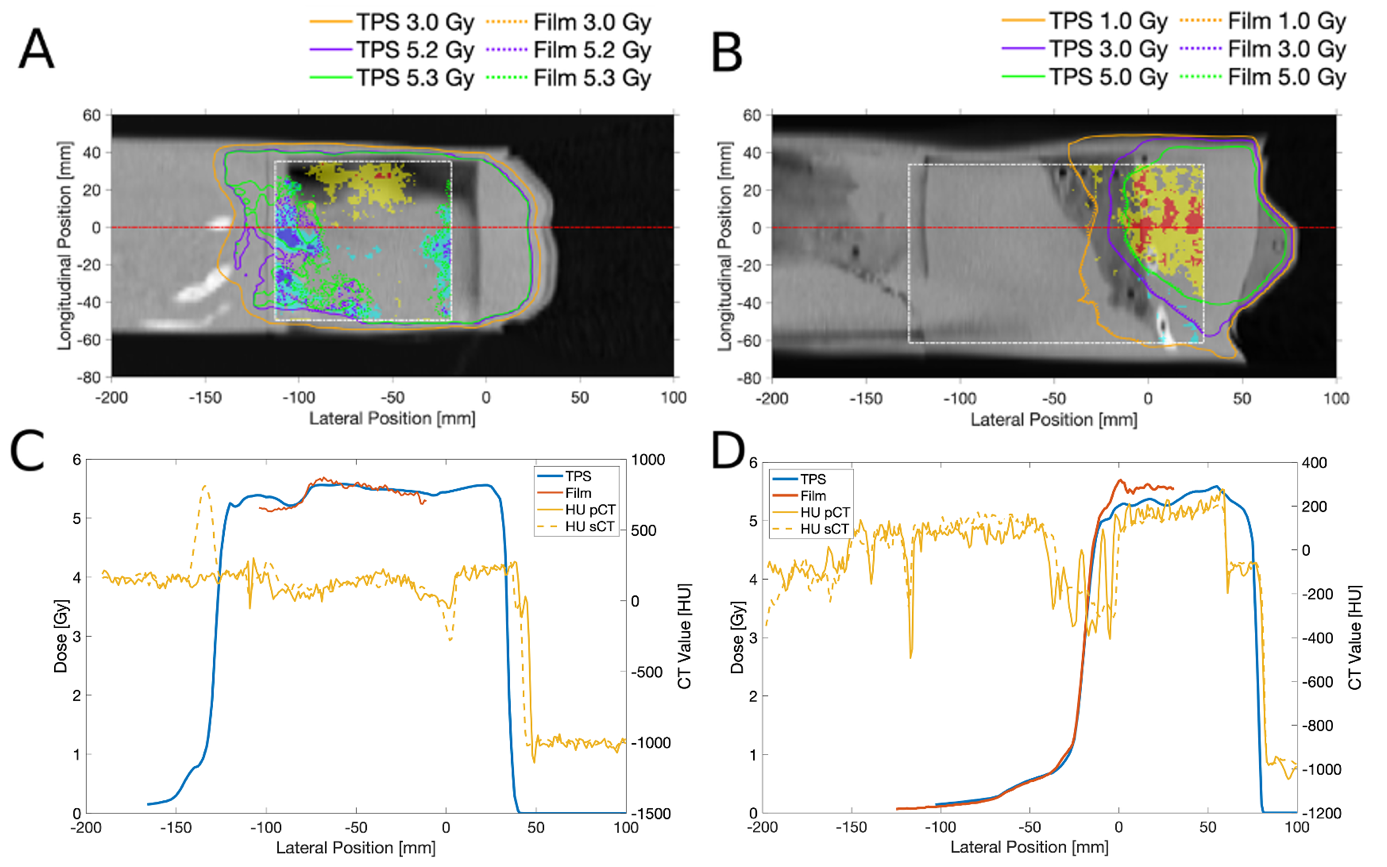


**Figure G12:** Gamma distribution and dose profiles for breast measurement B5 in plane 1 (A ,C) and plane 2 (B,D). (A,B) shows the film plane with overlayed isodose lines for measured and calculated dose in addition to the gamma index. Gamma is shown in yellow/red where the measured dose exceeds the calculated, and in cyan/blue where it falls below. The white rectangles in the gamma distribution represent the selected region of interest. (C,D) Dose profile graphs are shown for the red lines in (A,B), in addition to the HU-values for the pCT and sCT.


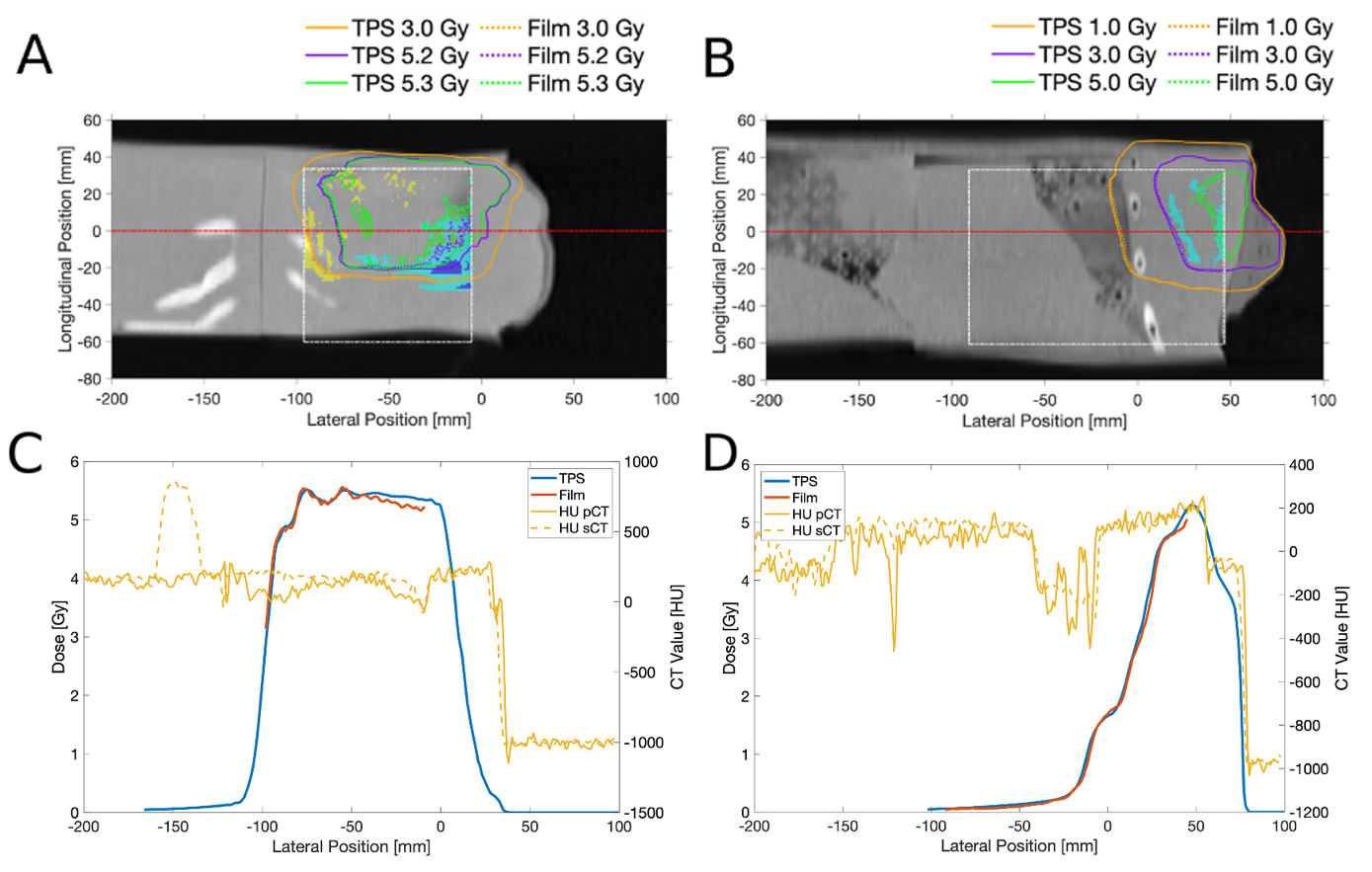


**Figure G13:** Gamma distribution and dose profiles for breast measurement B6 in plane 1 (A ,C) and plane 2 (B,D). (A,B) shows the film plane with overlayed isodose lines for measured and calculated dose in addition to the gamma index. Gamma is shown in yellow/red where the measured dose exceeds the calculated, and in cyan/blue where it falls below. The white rectangles in the gamma distribution represent the selected region of interest. (C,D) Dose profile graphs are shown for the red lines in (A,B), in addition to the HU-values for the pCT and sCT.


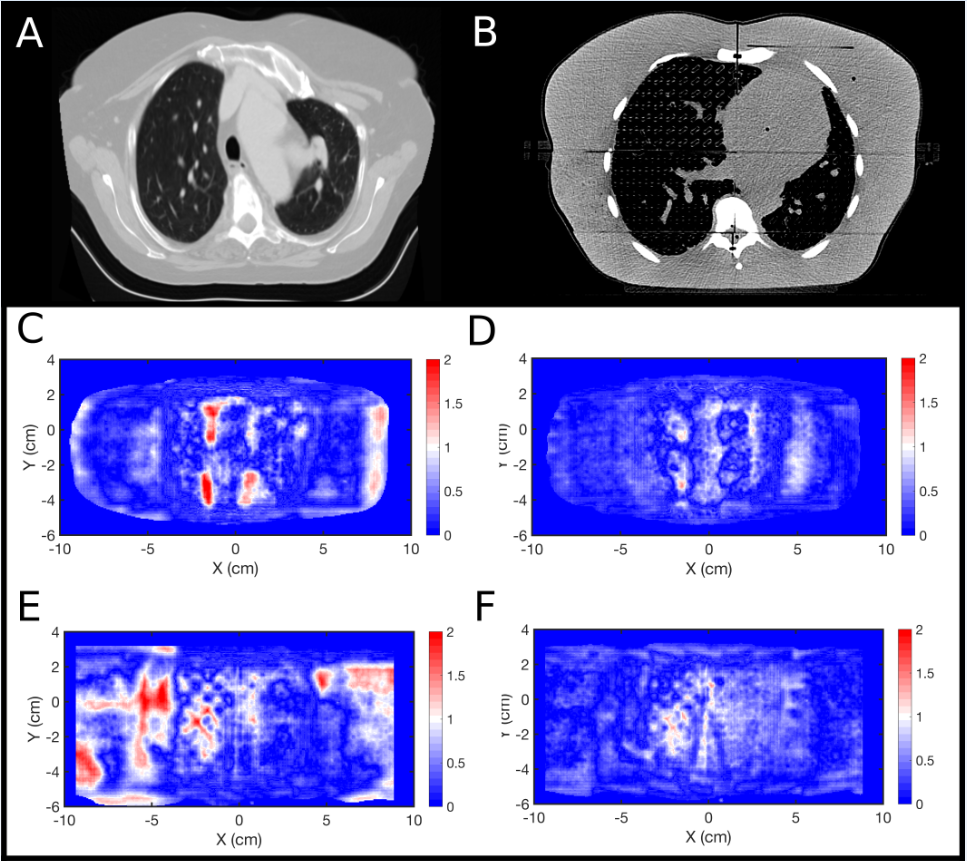
 **Figure G14:** Example dummy measurement spine: sCT and pCT for measurement S7 performed using a dummy patient CT, and their corresponding gamma distributions. C and D show the measurement plane in the spine, E and F show the measurement plane located in the lung.

**Supplementary section H: Gamma pass-rates for 3%/2mm**

**Table H1:** Gamma pass rates (GPR) for 3 %/2 mm analysis. CT: Computed Tomography, sCT: synthetic Computed Tomography.


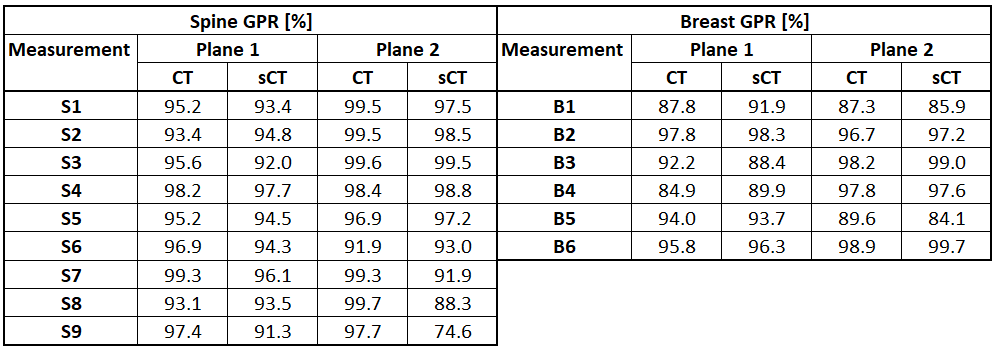

Supplement: Supplementary Data 1 [file mmc1.docx]
